# Molecular characteristics and outcomes in Hispanic and non‐Hispanic patients with acute myeloid leukemia

**DOI:** 10.1002/jha2.589

**Published:** 2022-10-18

**Authors:** Terrence Bradley, Deukwoo Kwon, Jorge Monge, Mikkael Sekeres, Namrata Chandhok, Amber Thomassen, Ronan Swords, Eric Padron, Jeff Lancet, Chetasi Talati, Justin Watts

**Affiliations:** ^1^ Division of Hematology, Sylvester Comprehensive Cancer Center University of Miami Miami Florida USA; ^2^ Division of Biostatistics, Sylvester Comprehensive Cancer Center University of Miami Miami Florida USA; ^3^ Division of Hematology and Medical Oncology Weill Cornell Medical College New York New York USA; ^4^ Division of Hematology and Medical Oncology Oregon Health Sciences University Portland Oregon USA; ^5^ Department of Malignant Hematology Moffitt Cancer Center Tampa Florida USA

**Keywords:** acute myeloid leukemia, ethnicity, Hispanic, molecular profile, outcomes

## Abstract

Hispanic patients have been reported to have an increased incidence of AML and possibly inferior outcomes compared to non‐Hispanics. We conducted a retrospective study of 225 AML patients (58 Hispanic and 167 non‐Hispanic) at two academic medical centers in Florida. Disease characteristics, cytogenetics, mutation profiles, and clinical outcomes were assessed. Hispanic patients were younger at presentation than non‐Hispanics (*p* = 0.0013). We found associations between single gene mutations and ethnicity, with IDH1 mutations being more common in non‐Hispanics (95.2% vs. 4.8%, *p* = 0.0182) and WT1 mutations more common in Hispanics (62.5% vs. 37.5%, *p* = 0.0455). We also found an emerging trend towards adverse risk cytogenetics in Hispanic patients (*p* = 0.1796), as well as high risk fusions such as MLL‐r (70% vs. 30%, *p* = 0.004). There was no difference in overall survival (OS) between Hispanic and non‐Hispanics patients. When examining only newly diagnosed patients (*n* = 105), there was improved OS in Hispanics (median 44.7 months vs. 14 months, *p* = 0.026) by univariate analysis and equivalent OS by multivariate analysis (hazard ratio = 1.52 [95% CI = 0.74–3.15]). Hispanics with a driver mutation not class‐defining had improved survival compared to non‐Hispanics. Our study demonstrates significant genetic differences between Floridian Hispanics and non‐Hispanics, but no difference in OS in patients treated at an academic medical center.

## 1 INTRODUCTION

Acute myeloid leukemia (AML) is a heterogeneous disorder characterized by clonal expansion of myeloid progenitor cells leading to uncontrolled immature myeloid cell proliferation and bone marrow failure. AML survival is mitigated by a number of known risk factors, including inherent disease pathobiology, clinical characteristics, and age and sociodemographic factors, leading to a 5‐year median overall survival for adult patients ranging from 5%–70%. Increasingly, outcome in myeloid malignancies, and even disease definition, has been linked to discrete molecular mutations.

Ethnic and racial disparities, attributable to both biologic and non‐biologic factors, have been described in several cancers, including AML. Compared to whites, for example, blacks have worse overall survival likely due to census tract disadvantage, affluence, and segregation (collectively called structural racism) and to certain biologic characteristics such as karyotype and molecular mutations. The Hispanic population comprises the largest minority group in the United States, currently representing nearly 20% of the population (26% in Florida), and it is expected to approach 30% by 2060. Cancer is the leading cause of death among Hispanics in the US (Prevention, CDC Fast Facts 2021; Arias et al National Vital Statistics 2021), who appear to develop distinct subtypes of leukemia: previous publications have revealed an increased incidence rate of B‐acute lymphoblastic leukemia (B‐ALL) and of acute promyelocytic leukemia (APL) [1, 2, 3, 4], which has been confirmed in an analysis of the Florida Cancer Data System.

Hispanics also may have an increased incidence rate of AML as a whole, possibly linked with inferior outcomes, compared to their non‐Hispanic counterparts [4]. This finding is supported by data on non‐APL AML from the Surveillance, Epidemiology, and End Results (SEER) database, which confirms a shorter median survival for Hispanic patients compared to non‐Hispanic white patients [5]. Whether differences in survival could be associated with distinct molecular mutations is unknown. To address this question, we assessed the molecular profiles and outcomes of Hispanics and non‐Hispanic patients with AML treated at two academic medical centers in Florida.

## 2 METHODS

### 2.1 Patients

Newly presenting patients diagnosed with AML per the 2016 World Health Organization at two cancer centers (Sylvester Comprehensive Cancer Center, University of Miami [SCCC/University of Miami (UM)] and the H. Lee Moffitt Cancer Center [MCC]) between the years of 2014 and 2017 (MCC 2014–2015 only) were included in this study, which was approved by Institutional Review Boards at both centers. Minimum age for inclusion was 18 years, and all newly presenting patients underwent next generation sequencing (NGS) using established 11‐ or 51‐gene myeloid panels performed by Genoptix Laboratories (now NeoGenomics Laboratories). Mutational profiling using an amplicon‐based targeted NGS platform was completed on an Illumina MiSeq instrument. All newly presenting AML patients had NGS performed as standard of care and were included in the analysis; no patients were excluded. Cytogenetic risk as defined per European LeukemiaNet criteria. Newly diagnosed patients were treated with less‐intensive and intensive chemotherapy regimens, with less‐intensive defined as hypomethylating agent‐based therapy, and more intensive as cytarabine‐ and anthracycline‐based regimens. Patients who had relapsed or refractory disease were also treated with less‐intensive and intensive therapies. Ethnicity (Hispanic or non‐Hispanic) was self‐identified by patients, as was race.

### 2.2 Statistics

Patient demographics and disease characteristics were summarized using descriptive statistics. The association between ethnicity and study covariates was examined using chi‐square test. For continuous variables, Wilcoxon signed‐rank test was used to compare groups. Overall survival analysis was measured from date of diagnosis until death due to any cause. The Kaplan–Meier method was used to derive survival curve estimates across groups, and the log‐rank test was used to comparisons survival curves. Univariable Cox proportional hazard regression model was performed to estimate hazard ratio (HR) and corresponding 95% confidence interval. Statistical significance was determined at a two‐sided alpha level of <0.05. False discovery rate method was used for multiplicity adjustment. Mutation plot was displayed along with key study covariates. Statistical analyses were carried out using SAS Software version 9.4 (SAS Institute Inc., Cary, NC) and R Software version 4.1.1.

## 3 RESULTS

### 3.1 Patient characteristics

Among 225 treated AML patients with targeted NGS performed, 58 (26%) were Hispanic, and 167 (74%) were non‐Hispanics Table 1); 96 patients (43%) were female. When examining race, 195 patients (87%) were white (147 non‐Hispanic and 48 Hispanic), 18 patients (8%) were black (17 African‐American and 1 Hispanic Black), three patients (1%) were Asian American (all non‐Hispanic), and nine patients (4%) were more than one race (all were Hispanic). The median age was 65 years (range 19–91). A total of 200 patients (89%) had an Eastern Cooperative Oncology Group (ECOG) score of 0–1, 17 (8%) of 2–3, and eight (4%) were missing.

**TABLE 1:** Patient demographics and disease characteristics

	All		Hispanic		Non‐Hispanic		
Variable	*N*	%	*N*	%	*N*	%	*p*‐Value
All	225	100.0	58	100.0	167	100.0	
Institution							0.020
Moffitt	83	36.9	14	24.1	69	41.3	
UM	142	63.1	44	75.9	98	58.7	
Gender							0.190
Female	96	42.7	29	50.0	67	40.1	
Male	129	57.3	29	50.0	100	59.9	
Race							<.0001
White	195	86.7	48	82.8	147	88.0	
Black	18	8.0	1	1.7	17	10.2	
Other	12	5.3	9	15.5	3	1.8	
Median age (range)	65 (19–91)		60 (21–85)		67 (19–91)		0.001
Age at diagnosis							0.019
≤60yos	88	39.1	31	53.4	57	34.1	
61–70yos	67	29.8	16	27.6	51	30.5	
>70yos	70	31.1	11	19.0	59	35.3	
ECOG							0.930
Missing	8	3.6	6	10.3	2	1.2	
ECOG:0	99	44.0	24	41.4	75	44.9	
ECOG:1‐3	118	52.4	28	48.3	90	53.9	
Subtype							0.003
2 more	4	1.8	2	3.4	2	1.2	
APL	1	0.4	1	1.7	.	.	
Driver not class‐defining	54	24.0	16	27.6	38	22.8	
IDH2 R172	1	0.4	1	1.7	.	.	
MLL/inv(3)/t(6;9)	10	4.4	7	12.1	3	1.8	
Mutated chromatin/splicing	48	21.3	9	15.5	39	23.4	
N/A	7	3.1	.	.	7	4.2	
NPM1/CEBPA/T(8;21)/inv(16)	33	14.7	4	6.9	29	17.4	
No driver	18	8.0	4	6.9	14	8.4	
TP53/aneuploidy	49	21.8	14	24.1	35	21.0	
Disease status							0.171
CR	19	8.4	8	13.8	11	6.6	
Newly Diagnosed	105	46.7	28	48.3	77	46.1	
Relapsed/Refractory	101	44.9	22	37.9	79	47.3	
Any mutations							0.554
No mutation	73	32.4	17	29.3	56	33.5	
One or more mutations	152	67.6	41	70.7	111	66.5	
Total number of mutations							
0	73	32.4	17	29.3	56	33.5	
1	85	37.8	28	48.3	57	34.1	
2	41	18.2	9	15.5	32	19.2	
3	19	8.4	4	6.9	15	9.0	
4	5	2.2	.	.	5	3.0	
5	2	0.9	.	.	2	1.2	
Overall survival							0.394
Alive	125	55.6	35	60.3	90	53.9	
Dead	100	44.4	23	39.7	77	46.1	

Abbreviations: APL, acute promyelocytic leukemia; CR, complete remission.

At time of NGS sample collection, 105 patients (47%) had newly diagnosed disease, 101 patients (45%) had relapsed/refractory disease, and 19 patients (8%) were in complete remission (Table 1). For newly diagnosed patients, the median WBC was 5.3 (range 0.3–238.4), and for relapsed/refractory patients it was 2.8 (range 0.2–312). Ninety (40%) of all patients, 17 (29.3%) Hispanics, and 73 (43.7%) non‐Hispanics had an antecedent hematologic disorder (*p* = 0.0762). The cytogenetic risk score in all patients was favorable in 7.6%, intermediate in 63.6%, adverse in 25.3%, and missing in 3.5%. The cytogenetic risk score in Hispanics was favorable (8.6%), intermediate (58.6%), and adverse (32.8%), and in non‐Hispanics it was favorable (7.2%), intermediate (65.3%), adverse (22.8%), and missing (4.8%). There was no difference in cytogenetic risk score between Hispanic and non‐Hispanic patients (*p* = 0.1796), although there was an emerging trend to adverse cytogenetic risk in Hispanics.

### 3.2 Mutations in Hispanics and non‐Hispanics

In the entire populations, 152 patients (68%) had a detectable mutation on the targeted NGS panel, 73 (32%) did not (Table 2). Consistent with other AML studies, the most common mutations were DNMT3A (18.2%), TET2 (17.8%), IDH2 (13.3%), ASXL1 (11.1%), FLT3 (7.1% ITD and 3.6% TKD), NPM1 (9.8%), and IDH1 (9.3%). In the 67 patients with an extended NGS panel performed, other mutations that occurred in over 5% of patients included RUNX1 (24%), SRSF2 (18%), TP53 (12%), and WT1 (12%), JAK2 (10%), NRAS (10%), STAG2 (9%), BCOR (9%), KRAS (9%), NF1 (9%), PDGFRB (8%), and SF3B1 (8%).

**TABLE 2:** Mutation frequency by ethnicity

	All		Hispanic		Non‐Hispanic		
Mutation	*N*	%	*N*	%	*N*	%	*p*‐Value*
All	225	100.0	58	100.0	167	100.0	
ASXL1							0.1434
No	200	88.9	55	94.8	145	86.8	
Yes	25	11.1	3	5.2	22	13.2	
CEBPA							1.00
No	195	91.1	48	90.6	147	91.3	
Yes	19	8.9	5	9.4	14	8.7	
DNMT3A							0.8456
No	184	81.8	47	81.0	137	82.0	
Yes	41	18.2	11	19.0	30	18.0	
IDH1							0.0182
No	204	90.7	57	98.3	147	88.0	
Yes	21	9.3	1	1.7	20	12.0	
IDH2							0.6541
No	195	86.7	49	84.5	146	87.4	
Yes	30	13.3	9	15.5	21	12.6	
KIT							0.4279
No	217	96.4	55	94.8	162	97.0	
Yes	8	3.6	3	5.2	5	3.0	
NPM1							0.4547
No	203	90.2	54	93.1	149	89.2	
Yes	22	9.8	4	6.9	18	10.8	
PHF6							1.00
No	207	92.0	54	93.1	153	91.6	
Yes	18	8.0	4	6.9	14	8.4	
TET2							1.00
No	185	82.2	48	82.8	137	82.0	
Yes	40	17.8	10	17.2	30	18.0	
FLT3‐TKD							0.6834
No	217	96.4	57	98.3	160	95.8	
Yes	8	3.6	1	1.7	7	4.2	
FLT3‐ITD							1.00
No	197	92.9	49	92.5	148	93.1	
Yes	15	7.1	4	7.6	11	6.9	
MLL‐PTD							0.3701
No	205	96.7	50	94.3	155	97.5	
Yes	7	3.3	3	5.7	4	2.5	
All	67	100.0	20	100.0	47	100.0	
BCOR							1.00
No	61	91.0	18	90.0	43	91.5	
Yes	6	9.0	2	10.0	4	8.5	
JAK2							0.6649
No	60	89.6	19	95.0	41	87.2	
Yes	7	10.4	1	5.0	6	12.8	
KRAS							0.0602
No	61	91.0	16	80.0	45	95.7	
Yes	6	9.0	4	20.0	2	4.3	
NF1							0.6604
No	61	91.0	19	95.0	42	89.4	
Yes	6	9.0	1	5.0	5	10.6	
NRAS							1.00
No	60	89.6	18	90.0	42	89.4	
Yes	7	10.4	2	10.0	5	10.6	
PDGFRB							0.6306
No	62	92.5	18	90.0	44	93.6	
Yes	5	7.5	2	10.0	3	6.4	
RUNX1							0.7597
No	51	76.1	16	80.0	35	74.5	
Yes	16	23.9	4	20.0	12	25.5	
SF3B1							1.00
No	62	92.5	19	95.0	43	91.5	
Yes	5	7.5	1	5.0	4	8.5	
SRSF2							0.7410
No	55	82.1	16	80.0	39	83.0	
Yes	12	17.9	4	20.0	8	17.0	
STAG2							0.1678
No	61	91.0	20	100.0	41	87.2	
Yes	6	9.0	*	*	6	12.8	
TP53							0.6872
No	59	88.1	17	85.0	42	89.4	
Yes	8	11.9	3	15.0	5	10.6	
WT1							0.0455
No	59	88.1	15	75.0	44	93.6	
Yes	8	11.9	5	25.0	3	6.4	
KRAS, NRAS, and NF1							0.2756
No	206	91.6	51	92.8	155	87.9	
Yes	19	8.4	7	7.2	12	12.1	
SF3B1, SRSF2, U2AF1, or ZRSR2							1.00
No	48	71.6	14	70.0	34	72.3	
Yes	19	28.4	6	30.0	13	27.7	

* *p*‐Values were obtained from chi‐square test.

Focusing on mutation differences between Hispanics and non‐Hispanics, for discrete mutations, rates were similar between Hispanics and non‐Hispanics. IDH1 mutations, but not IDH2, were significantly more common in non‐Hispanic versus Hispanic patients (95.2% vs. 4.8%, *p* = 0.0182) (Table 2, Figure S1). However, after adjusting multiple testing, *p*‐value was no longer significant (*p* = 0.218). On the other hand, WT1 mutations were more common in Hispanic patients compared to non‐Hispanics (62.5% vs. 37.5%, *p* = 0.0455), with an adjusted *p*‐value of 0.421.

The total number of mutations in Hispanics appeared to be less, with a mean number of 1, compared to a mean number of 1.17 for non‐Hispanics (relative risk 0.85 [95% CI = 0.64–1.14; *p* = 0.284]) by univariate analysis (Figure S2). When we performed multivariate analysis for age along with Hispanic status the relative risk for Hispanic was 0.90 (95% CI = 0.66–1.23; *p* = 0.496).

We also examined for associations between ethnicity and mutation class/molecular subgroups [6]. The most common mutation classes were driver not class defining (24%), TP53/aneuploidy (22%), and mutated chromatin/splicing (21%) (Table 3). Favorable mutations NPM1/CEBPA/t(8;21)/inv(16) were observed in 18% of patients. There was a trend toward non‐Hispanics being more likely to have a favorable mutation compared to Hispanics at 12.1% versus 87.9%, respectively (*p* = 0.084). Adverse risk fusions MLL/inv(3)/t(6;9) were seen in 4.4% of patients and, although rare, were more common in Hispanic patients compared to non‐Hispanics (70% vs. 30%, *p* = 0.004), and this remained significant after multiplicity adjustment (*p* = 0.024).

**TABLE 3:** AML molecular subgroups by ethnicity

	All		Hispanic		Non‐Hispanic		
Variable	*N*	%	*N*	%	*N*	%	*p*‐Value
All	225	100.0	58	100.0	167	100.0	
Subtype							
2 more	4	1.8	2	3.4	2	1.2	‐
APL	1	0.4	1	1.7	.	.	‐
Driver not class‐defining	54	24.0	16	27.6	38	22.8	0.573
IDH2 R172	1	0.4	1	1.7	.	.	‐
MLL‐r/inv(3)/t(6;9)	10	4.4	7	12.1	3	1.8	0.004
Mutated chromatin/splicing	48	21.3	9	15.5	39	23.4	0.285
N/A	7	3.1	.	.	7	4.2	‐
NPM1/CEBPA/t(8;21)/inv(16)	33	14.7	4	6.9	29	17.4	0.084
No driver	18	8.0	4	6.9	14	8.4	0.937
TP53/aneuploidy	49	21.8	14	24.1	35	21.0	0.748

### 3.3 Patient outcomes

#### 3.3.1 Full Cohort

**FIGURE 1:**
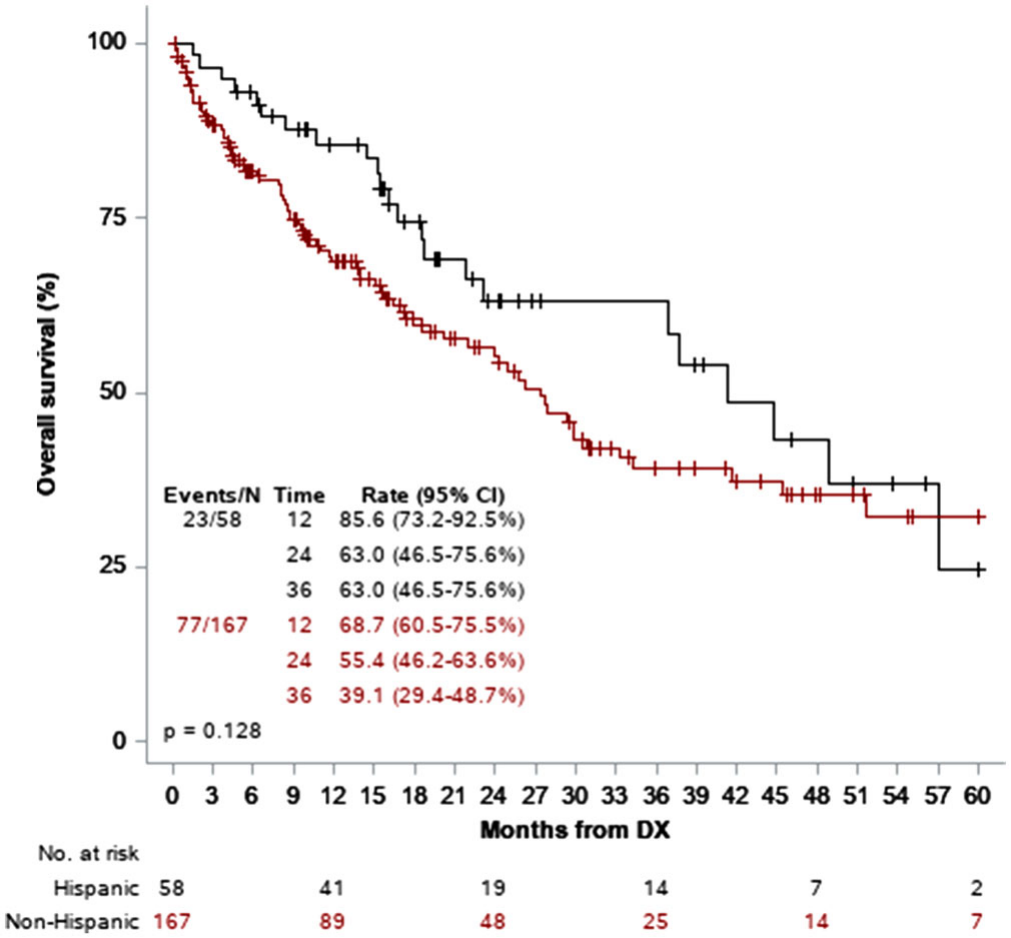
Overall survival by ethnicity in all patients

With a median follow‐up of 24.4 months (range, 0.2–147), 125 patients were still alive (55.6%): 35 Hispanic patients (60.3%) and 90 non‐Hispanic patients (53.9%). There was no difference in follow‐up between the two groups (*p* = 0.443). There was no difference in OS between Hispanics and non‐Hispanics (*p* = 0.128, Figure 1), although numerically Hispanics had improved OS at 12, 24, and 36 months, with the curves converging at 48 months. The 1‐year OS for Hispanic patients was 85.6% and for non‐Hispanic patients was 68.7% (*p* = 0.589), and 3‐year OS for Hispanic patients was 63.0% versus 39.1% for non‐Hispanic patients (*p* = 0.735). The univariate HR for survival for non‐Hispanics was 1.43 (95% CI = 0.9–2.29; *p* = 0.13). In multivariate analysis considering age, sex, ECOG, cytogenetic risk, and institution, the HR for survival for non‐Hispanics was 1.15 (95% CI = 0.69–1.90; *p* = 0.594). Sixteen Hispanic patients (27.6%) underwent allogeneic hematopoietic stem cell translation, compared to 36 non‐Hispanic patients (21.7%, *p* = 0.37).

#### 3.3.2 De novo patients

**FIGURE 2:**
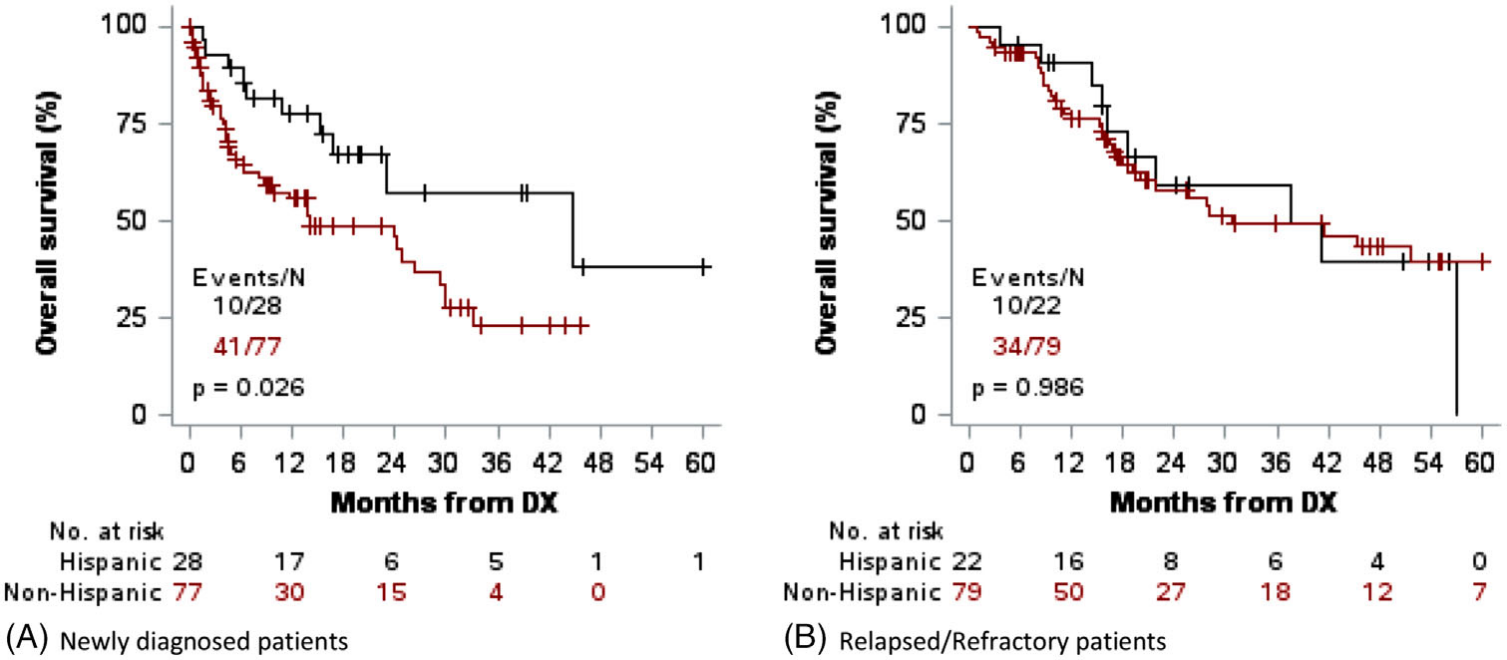
Overall survival in newly diagnosed patients and relapsed/refractory patients

In patients who were newly diagnosed (105 patients), OS was significantly better for Hispanic patients compared to non‐Hispanics, at a median of 44.7 months versus 14 months (log‐rank test *p*‐value = 0.026; HR = 0.46 [95% CI = 0.23–0.92], *p* = 0.0292, Figure 2), which is partially explained by Hispanics being younger at diagnosis than non‐Hispanics (all patients, *p* = 0.0013; newly diagnosed group, *p* = 0.0529). In multivariate analysis accounting for age, sex, ECOG, and treating institution, this difference in survival persisted, but was no longer significant (HR = 0.66 [95% CI = 0.32–1.35], *p* = 0.2573).

#### 3.3.3 R/R patients

Among the 101 patients with relapsed/refractory disease, there was no difference in OS by univariate or multivariate analysis (median 37.7 months for Hispanics, 30.9 months for non‐Hispanics, HR = 0.99 [95% CI = 0.49–2.01], *p* = 0.986 for UVA and HR = 0.97 [95% CI = 0.45–2.09], *p* = 0.933 for MVA, Figure 2).

### 3.4 Patient outcomes by molecular subgroups

**FIGURE 3:**
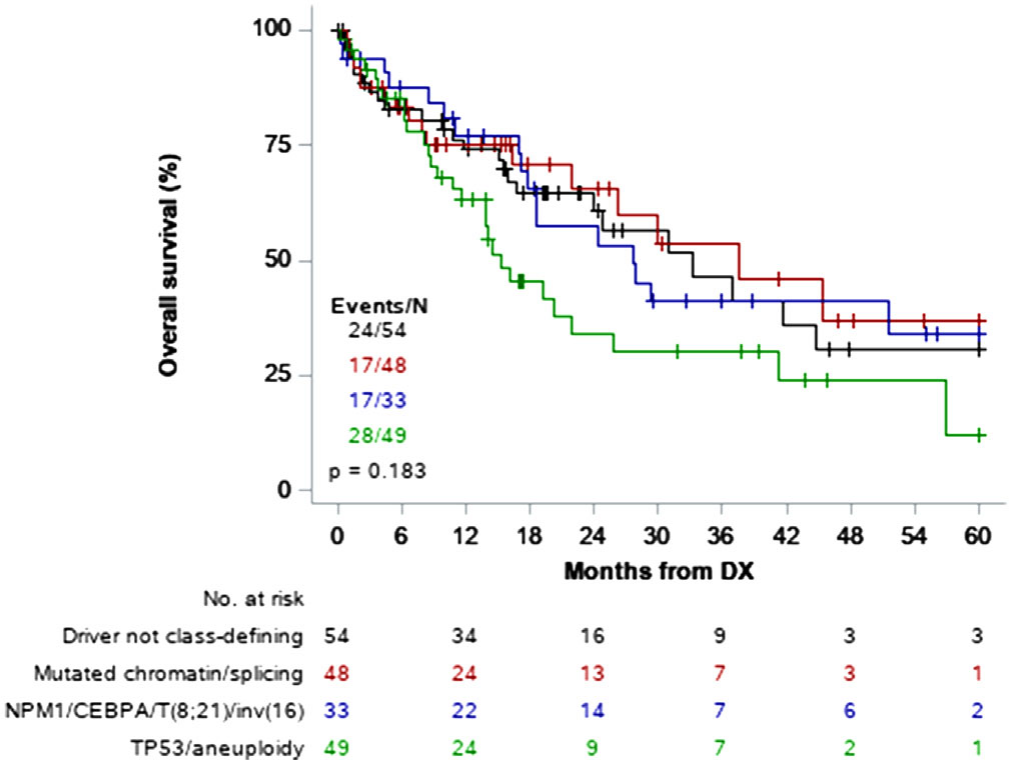
Overall survival by molecular subgroup

**FIGURE 4:**
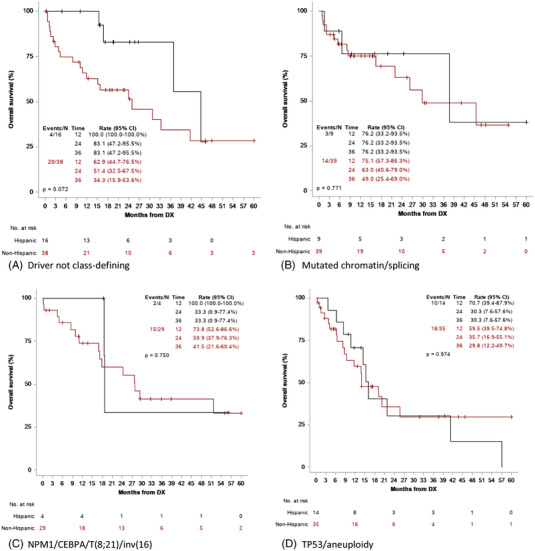
Overall survival for molecular subgroups by ethnicity

We also examined OS by the most common class‐defining molecular subgroups: driver not class‐defining, mutated chromatin/splicing, NPM1/CEBPA/t(8;21)/inv(16), and TP53/aneuploidy [6]. Patients with TP53/aneuploidy had inferior OS compared to the other three mutation sub‐categories (*p* = 0.183) (Figure 3). There was no difference in survival in Hispanic versus non‐Hispanic patients with TP53/aneuploidy, mutated chromatin splicing, or NPM1/CEBPA/t(8;21)/inv(16) (*p* = 0.943) (Figure 4). However, Hispanic patients with driver not class‐defining mutations had improved OS at 100% versus 62.9% at 1 year (*p* < .0001) and 83.1% versus 34.3% at 3 years (*p* = 0.072) (Figure 4). In the driver not class‐defining group, there were 14 total patients with IDH mutations, 12 non‐Hispanic and two Hispanic (*p* = 0.008). The two IDH mutations occurring in Hispanic patients were both IDH2 R140. When we examined splicing factor mutations as a group (SF3B1, SRSF2, U2AF1, and ZRSR2), while the frequency was not different by ethnicity, OS was significantly better in non‐Hispanic patients (*p* = 0.010) (Figure S3). The frequency of RAS pathway mutations between ethnicity groups was also not different, although OS favored non‐Hispanic patients (*p* = 0.057) (Figure S4).

## 4 DISCUSSION

From a healthcare perspective, racial and ethnic minorities in the United States broadly encompass Hispanics, African Americans or Blacks, American Indians and Alaska Natives, Asians, Native Hawaiians and Pacific Islanders according to the National Institute on Minority Health and Health Disparities [7]. The Hispanic population collectively constitutes the largest minority group in the United States and currently represents approximately 18% of the US population [3, 4]. This group includes people of Mexican, South/Central American, Cuban, Puerto Rican, or other Spanish‐speaking cultures with half of the current Hispanic population residing in three states: Florida, Texas, and California [8, 9]. There are data to suggest that while the incidence of AML, barring APL, is lower in Hispanics compared to non‐Hispanics, they are often diagnosed at a younger age and may have a poorer age adjusted overall survival [5, 10–13]. The genetic and socioeconomic differences accounting for the possible discrepancies in prognosis and survival are not well accounted for at this time.

To better answer these questions, we examined NGS data on 225 AML patients and found that certain gene mutations clustered by ethnicity. IDH1 mutations were more common in non‐Hispanic patients, while WT1 mutations were more common in Hispanic patients. Interestingly, both of these genes affect TET protein activity and DNA methylation. However, there was no association between TET2 or IDH2 mutations, or any other single gene mutation, and ethnicity. We also examined mutation risk groups/molecular subgroups and found that Hispanic patients were more likely to have an adverse risk AML fusion [11q23, inv(3), t(6;9)], and non‐Hispanic patients were more likely to have a favorable risk fusion/mutation [t(8;21), inv(16), NPM1, CEBPA]. The strongest association was between Hispanic ethnicity and adverse risk AML fusions, which remained statistically significant after multiplicity adjustment (adjusted *p*‐value = 0.024). FLT3‐ITD and TKD mutations were underrepresented in this cohort for unclear reasons. In non‐Hispanics this may have been due to older age at presentation. In Floridian Hispanics FLT3 mutations were relatively uncommon (ITD in 7.6% and TKD in 1.7%).

We acknowledge that our study included all newly presenting AML patients, and that 19 (8.4%) of these patients were in complete remission after initially receiving treatment at outside centers. These patients were included in analysis of mutations since pre‐leukemic mutations often persist in remission. However, driver mutations may have been missed in these patients and decreased the rate of detection of NPM1 and FLT3 mutations, for example.

Interestingly, Hispanic patients had improved OS compared to non‐Hispanics, although the curves converged over time at around 4 years (*p* = 0.128). When we examined only newly diagnosed patients, Hispanic patients had a significant and lasting improvement in OS (*p* = 0.026). These observations are despite Hispanic patients appearing to be enriched for prognostically adverse cytogenetic abnormalities, particularly high‐risk fusions such as mixed lineage leukemia re‐arrangement (MLL‐r). When we performed multivariate analysis accounting for age, sex, ECOG, cytogenetic risk, and treating institution, this difference in OS was no longer observed, likely due to Hispanics being younger at time of AML diagnosis than non‐Hispanics. Whether this age difference at AML diagnosis is due to the demographics of Florida versus differences in risk factors or disease biology remains to be discovered. Hispanic patients were also more likely to have de novo rather than secondary AML. At the minimum, and contrary to prior reports, our study suggests that Hispanic patients have *equivalent* age‐adjusted OS compared to non‐Hispanics, particularly if they are treated at an academic medical center. This may be due to the equalizing of socioeconomic disparities that can contribute to more advanced presentation, inadequate follow‐up, and lack of availability of allogeneic stem cell transplantation. There was no difference in the rate of allogeneic transplantation in different ethnic groups on our study.

We sought to further assess survival differences in Hispanics and non‐Hispanics with AML by examining distinct, class‐defining molecular subgroups. In the entire cohort, patients with TP53/aneuploidy, regardless of ethnicity, had the poorest outcomes, although there was a tail on the curve with 10%–15% long‐term survival. Interestingly, Hispanic patients in the driver not‐class defining category had improved OS relative to non‐Hispanics. One possible explanation for this difference is that non‐Hispanic patients were enriched for mutations involved in cellular metabolism, suggesting that the heterogeneous driver not‐class defining category could be sub‐divided by the presence or absence of mutations in IDH1/2. Hispanic patients did not have inferior OS in any molecular subgroup. However, when we looked at specific mutation classes, Hispanic patients with RAS pathway or RNA splicing factor mutations had inferior OS compared to non‐Hispanics (*p* = 0.057 and *p* = 0.010, respectively).

This work sought to identify biologic discrepancies between Hispanic and non‐Hispanic patients diagnosed with AML, which is not well characterized. Data from a Mexican AML population suggested, CEBPA, RUNX1 GATA2, TET2, AML1‐ETO, U2AF1, ASXL1, and KIT are enriched in that population, whereas FLT3‐ITD, DNMT3A, NPM1, and IDH2 were under‐represented [14]. Our data of Floridian patients collected at two large academic centers showed two stand out differences on NGS; IDH1 mutations were underrepresented, and WT1 mutations were enriched in Hispanic patients compared to non‐Hispanics, with a possibly lower overall mutational burden in Hispanic AML patients (possibly explained by younger age at presentation). Looking at mutation groups, there was a trend toward more adverse risk fusions and fewer favorable risk mutations in Floridian Hispanics; however, the rate of secondary AML was higher in non‐Hispanics (which may be associated with age at presentation). In terms of the impact of specific mutation groups on outcomes, Hispanics patients without a Class defining driver mutation had improved OS compared to non‐Hispanics, while those with a RAS pathway or splicing factor mutation had inferior OS.

In this study, after adjustment for age and other confounders, there was no significant difference in OS between Hispanic and non‐Hispanic patients. That is to say, *when receiving equivalent care at an academic cancer center, overall survival may be comparable between Hispanic and Non‐Hispanic patients, despite any molecular differences associated with ethnicity*.

There are some limitations to this study. Sample size in this study is limited and only includes those who geographically, and financially, could seek care at an academic center. This may in turn inherently exclude those with the worst SES and everything that comes along with it such as environmental exposures and other health stressors. Prior work as shown that regional location can play a role in AML outcomes in the Hispanic population [15]. Factors such as poverty, income, and education status have also been linked to inferior survival, which may explain the inferior risk reported in SEER Medicare analysis [5, 12, 16–18]. Our data may suggest that, while there are some genetic differences, “modifiable” factors such as socioeconomic inequity, healthcare access, environmental factors, etc. are the primary drivers for the discrepancies in outcome reported in the past, and correction of these factors could improve AML outcomes in Hispanic patients. Secondly, the Hispanic diaspora is diverse and includes people of Mexican, South/Central American, Cuban, Puerto Rican, or other Spanish‐speaking cultures. Ethnicity on this study was self‐reported, so we do not know the exact descent, which is quite vast for Hispanics and may drive genetic differences. While Florida is one of three states where half the Hispanic population currently resides (the others being California and Texas), it likely over‐represents certain subsets of the Hispanic population, such as Cuban or Puerto Rican Americans who are genetically disparate from other under‐represented groups, such as those of Mexican origin [10, 11]. Strengthening these data to further characterize the relationship between ethnicity and AML genotype and outcomes will require a multi‐institutional effort moving forward.

## AUTHOR CONTRIBUTIONS

TB, JM, and CT collected and analyzed the data, interpreted the data, and edited the manuscript. DK performed statistical analysis and edited the manuscript. AT, MS, NC, RS, EP, and JL analyzed and interpreted the data and edited the manuscript. JW conceived and coordinated the study, analyzed and interpreted the data, and wrote the manuscript. All authors discussed the results and commented on the manuscript.

## FUNDING INFORMATION

National Institutes of Health, Grant Number: P30 CA008748

## CONFLICT OF INTEREST

The authors declare that there is no conflict of interest that could be perceived as prejudicing the impartiality of the research reported.

## ETHICS STATEMENT

This study was IRB‐approved under protocol 20160255 on April 14, 2016. The IRB approved the study with a waiver of consent (retrospective chart review).

## Supporting information

Supporting Information

## Data Availability

The data that support the findings of this study are available from the corresponding author upon reasonable request.
