# Supplementary material for: Molecular characteristics and outcomes in Hispanic and non‐Hispanic patients with acute myeloid leukemia
Source: EJHaem. 2022 Oct 18;3(4):1231–40. doi: 10.1002/jha2.589 (PMC9713060; doi:10.1002/jha2.589)
Supplement: Supplementary file 1 — Supporting Information [file JHA2-3-1231-s001.pdf]

eJHaem British Society for Haematology Listening • Learning • Leading

Molecular Characteristics and Outcomes in Hispanic and Non-Hispanic Patients with Acute Myeloid Leukemia

Journal:	*eJHaem*
Manuscript ID	ejhaem-2022-09-0689
Wiley - Manuscript type:	Research Article
Date Submitted by the Author:	16-Sep-2022
Complete List of Authors:	Monge, Jorge; Weill Cornell Medicine Department of Hematology and Oncology, Hematology and Oncology<br>Thomassen, Amber; University of Miami School of Medicine,<br>Padron, Eric; Malignant Hematology, H. Lee Moffitt Cancer Center and Research Institute, Tampa, FL, USA<br>Lancet, Jeffrey; H Lee Moffitt Cancer Center and Research Institute, Department of Malignant Hematology<br>Watts, Justin; University of Miami Miller School of Medicine<br>Bradley, Terrence J ; Sylvester Comprehensive Cancer Center, Malignant Hematology<br>Kwon, Deukwoo; University of Miami Miller School of Medicine, Biostatistics<br>Sekeres, Mikkael; University of Miami, Hematology/ Sylvester Comprehensive Cancer Center<br>Talati, Chetasi; AbbVie Inc, Hematology; Moffitt Cancer Center, Hematology
Key Words:	Hispanic, AML, MOLECULAR ANALYSIS
Abstract:	Hispanic patients have been reported to have an increased incidence of AML and possibly inferior outcomes compared to non-Hispanics. We conducted a retrospective study of 225 AML patients (58 Hispanic and 167 non-Hispanic) at two academic medical centers in Florida. Disease characteristics, cytogenetics, mutation profiles, and clinical outcomes were assessed. Hispanic patients were younger at presentation than non-Hispanics (p=0.0013). We found associations between single gene mutations and ethnicity, with IDH1 mutations being more common in non-Hispanics (95.2% vs. 4.8%, p=0.0182) and WT1 mutations more common in Hispanics (62.5% vs. 37.5%, p=0.0455). We also found an emerging trend towards adverse risk cytogenetics in Hispanic patients (p=0.1796), as well as high risk fusions such as MLL-r (70% vs. 30%, p=0.004). There was no difference in overall survival (OS) between Hispanic and non-Hispanics patients. When examining only newly diagnosed patients (n=105), there was improved OS in Hispanics (median 44.7 months vs. 14 months, p=0.026) by univariate analysis and equivalent OS by multivariate analysis (HR=1.52 [95% CI=0.74-3.15]). Hispanics with a driver mutation not class-defining had improved survival compared to non-Hispanics. Our study demonstrates significant genetic differences between Floridian Hispanics and non-Hispanics, but no difference in OS in patients treated at an academic medical center.

SCHOLARONE™
Manuscripts

Molecular Characteristics and Outcomes in Hispanic and Non-Hispanic Patients with Acute Myeloid Leukemia

Running title: Ethnic differences in mutation profiles and outcomes in AML

Terrene Bradley[1], Deukwoo Kwon[2], Jorge Monge[3], Mikkael Sekeres[1], Namrata Chandhok[1], Amber Thomassen[1], Ronan Swords[4], Eric Padron[5], Jeff Lancet[5], Chetasi Talati[5], Justin Watts[1]

Author affiliations:

[1]University of Miami, Division of Hematology, Sylvester Comprehensive Cancer Center, Miami, FL, USA; [2]University of Miami, Division of Biostatistics, Sylvester Comprehensive Cancer Center, Miami, FL, USA; [3]Weill Cornell Medical College, Division of Hematology and Medical Oncology, New York, NY, 10065; [4]Oregon Health Sciences University, Division of Hematology and Medical Oncology, Portland, OR, 97239; [5]Moffitt Cancer Center, Department of Malignant Hematology, Tampa, FL, 33612

Corresponding author:

Justin M. Watts, MD

University of Miami

Sylvester Comprehensive Cancer Center

1121 NW 14th Street (SMOB), 210B

Miami, FL 33136, USA

Tel: +1-305-243-8986

Fax: +1-305-243-9161

Email: jxw401@miami.edu

Data sharing: For original data, please contact jxw401@miami.edu.

Abstract word count: 200

Total word count: 2921

Figures and Tables: 7 (4 figures and 3 tables)

Supplemental files: 4 figures

For Review Only

Acknowledgements: This work was supported by a National Institutes of Health P30 CA008748 – Cancer Center Support Grant.

Disclosures: No conflicts of interest to disclose.

Contributions: TB, JM, and CT collected and analyzed the data, interpreted the data, and edited the manuscript. DK performed statistical analysis and edited the manuscript. AT, MS, NC, RS, EP, and JL analyzed and interpreted the data and edited the manuscript. JW and conceived and coordinated the study, analyzed and interpreted the data, and wrote the manuscript. All authors discussed the results and commented on the manuscript.

Abstract

Hispanic patients have been reported to have an increased incidence of AML and possibly inferior outcomes compared to non-Hispanics. We conducted a retrospective study of 225 AML patients (58 Hispanic and 167 non-Hispanic) at two academic medical centers in Florida. Disease characteristics, cytogenetics, mutation profiles, and clinical outcomes were assessed. Hispanic patients were younger at presentation than non-Hispanics (p=0.0013). We found associations between single gene mutations and ethnicity, with IDH1 mutations being more common in non-Hispanics (95.2% vs. 4.8%, p=0.0182) and WT1 mutations more common in Hispanics (62.5% vs. 37.5%, p=0.0455). We also found an emerging trend towards adverse risk cytogenetics in Hispanic patients (p=0.1796), as well as high risk fusions such as MLL-r (70% vs. 30%, p=0.004). There was no difference in overall survival (OS) between Hispanic and non-Hispanics patients. When examining only newly diagnosed patients (n=105), there was improved OS in Hispanics (median 44.7 months vs. 14 months, p=0.026) by univariate analysis and equivalent OS by multivariate analysis (HR=1.52 [95% CI=0.74-3.15]). Hispanics with a driver mutation not class-defining had improved survival compared to non-Hispanics. Our study demonstrates significant genetic differences between Floridian Hispanics and non-Hispanics, but no difference in OS in patients treated at an academic medical center.

For Review Only

Introduction

Acute myeloid leukemia (AML) is a heterogeneous disorder characterized by clonal expansion of myeloid progenitor cells leading to uncontrolled immature myeloid cell proliferation and bone marrow failure. AML survival is mitigated by a number of known risk factors, including inherent disease pathobiology, clinical characteristics, and age and sociodemographic factors, leading to a 5-year median overall survival for adult patients ranging from 5-70%. Increasingly, outcome in myeloid malignancies, and even disease definition, has been linked to discrete molecular mutations.

Ethnic and racial disparities, attributable to both biologic and non-biologic factors, have been described in several cancers, including AML. Compared to whites, for example, blacks have worse overall survival likely due to census tract disadvantage, affluence, and segregation (collectively called structural racism) and to certain biologic characteristics such as karyotype and molecular mutations. The Hispanic population comprises the largest minority group in the United States, currently representing nearly 20% of the population (26% in Florida), and it is expected to approach 30% by 2060. Cancer is the leading cause of death among Hispanics in the US (Prevention, CDC Fast Facts 2021; Arias et al National Vital Statistics 2021), who appear to develop distinct subtypes of leukemia: previous publications have revealed an increased incidence rate of B-acute lymphoblastic leukemia (B-ALL) and of acute promyelocytic leukemia (APL)[1-4]which has been confirmed in an analysis of the Florida Cancer Data System (FCDS).

Hispanics also may have an increased incidence rate of AML as a whole, possibly linked with inferior outcomes, compared to their non-Hispanic counterparts[4] This finding is supported by data on non-APL AML from the Surveillance, Epidemiology, and End Results (SEER) database, which confirms a shorter median survival for Hispanic patients compared to non- Hispanic white patients[5]. Whether differences in survival could be associated with distinct molecular mutations is unknown. To address this question, we assessed the molecular profiles and outcomes of Hispanics and non-Hispanic patients with AML treated at 2 academic medical centers in Florida.

Methods

Patients

Newly presenting patients diagnosed with AML per the 2016 World Health Organization at two cancer centers (Sylvester Comprehensive Cancer Center, University of Miami [SCCC] and the H. Lee Moffitt Cancer Center [MCC]) between the years of 2014 and 2017 (MCC 2014-2015 only) were included in this study, which was approved by Institutional Review Boards at both centers. Minimum age for inclusion was 18 years, and all newly presenting patients underwent next generation sequencing (NGS) using

For Review Only

established 11- or 51-gene myeloid panels performed by Genoptix Laboratories (now NeoGenomics Laboratories). Mutational profiling using an amplicon-based targeted NGS platform was completed on an Illumina MiSeq instrument. All newly presenting AML patients had NGS performed as standard of care and were included in the analysis; no patients were excluded. Cytogenetic risk as defined per European LeukemiaNet criteria. Newly diagnosed patients were treated with less-intensive and intensive chemotherapy regimens, with less-intensive defined as hypomethylating agent-based therapy, and more intensive as cytarabine- and anthracycline-based regimens. Patients who had relapsed or refractory disease were also treated with less-intensive and intensive therapies. Ethnicity (Hispanic or non-Hispanic) was self-identified by patients, as was race.

Statistics

Patient demographics and disease characteristics were summarized using descriptive statistics. The association between ethnicity and study covariates were examined using Chi-square test. For continuous variables, Wilcoxon signed- rank test was used to compare groups. Overall survival analysis was measured from date of diagnosis until death due to any cause. The Kaplan-Meier method was used to derive survival curve estimates across groups, and the log-rank test was used to comparisons survival curves. Univariable Cox proportional hazard regression model was performed to estimate hazard ratio (HR) and corresponding 95% confidence interval. Statistical significance was determined at a two-sided alpha level of <0.05. False discovery rate (FDR) method was used for multiplicity adjustment. Mutation plot was displayed along with key study covariates. Statistical analyses were carried out using SAS Software version 9.4 (SAS Institute Inc., Cary, NC) and R Software version 4.1.1.

For Review Only

Results

Patient Characteristics

Among 225 treated AML patients with targeted NGS performed, 58 (26%) were Hispanic and 167 (74%) were non-Hispanics Table 1); 96 patients (43%) were female. When examining race, 195 patients (87%) were white (147 non-Hispanic and 48 Hispanic), 18 patients (8%) were black (17 African-American and 1 Hispanic Black), 3 patients (1%) were Asian American (all non-Hispanic), and 9 patients (4%) were more than one race (all were Hispanic). The median age was 65 years (range 19-91). A total of 200 patients (89%) had an ECOG score of 0-1, 17 (8%) of 2-3, and 8 (4%) were missing.

At time of NGS sample collection, 105 patients (47%) had newly diagnosed disease, 101 patients (45%) had relapsed/refractory disease, and 19 patients (8%) were in complete remission (CR) (Table 1). For newly diagnosed patients, the median WBC was 5.3 (range 0.3-238.4) and for relapsed/refractory patients it was 2.8 (range 0.2-

312). Ninety (40%) of all patients, 17 (29.3%) Hispanics, and 73 (43.7%) non-Hispanics had an antecedent hematologic disorder (p=0.0762). The cytogenetic risk score in all patients was favorable in 7.6%, intermediate in 63.6%, adverse in 25.3%, and missing in 3.5%. The cytogenetic risk score in Hispanics was favorable (8.6%), intermediate (58.6%), and adverse (32.8%), and in non-Hispanics it was favorable (7.2%), intermediate (65.3%), adverse (22.8%), and missing (4.8%). There was no difference in cytogenetic risk score between Hispanic and non-Hispanic patients (p=0.1796), although there was an emerging trend to adverse cytogenetic risk in Hispanics.

Mutations in Hispanics and Non-Hispanics

In the entire populations, 152 patients (68%) had a detectable mutation on the targeted NGS panel, 73 (32%) did not. (Table 2). Consistent with other AML studies, the most common mutations were DNMT3A (18.2%), TET2 (17.8%), IDH2 (13.3%), ASXL1 (11.1%), FLT3 (7.1% ITD and 3.6% TKD), NPM1 (9.8%), and IDH1 (9.3%). In the 67 patients with an extended NGS panel performed, other mutations that occurred in over 5% of patients included RUNX1 (24%), SRSF2 (18%), TP53 (12%), and WT1 (12%), JAK2 (10%), NRAS (10%), STAG2 (9%), BCOR (9%), KRAS (9%), NF1 (9%), PDGFRB (8%), and SF3B1 (8%).

Focusing on mutation differences between Hispanics and non-Hispanics, for discrete mutations, rates were similar between Hispanics and non-Hispanics. IDH1 mutations, but not IDH2, were significantly more common in non-Hispanic vs. Hispanic patients (95.2% vs. 4.8%, p=0.0182) [Table 2, Suppl Fig 1]. However, after adjusting multiple testing, p-value was no longer significant (p=0.218). On the other hand, WT1 mutations were significantly more common in Hispanic patients compared to non-Hispanics (62.5% vs. 37.5%, p=0.0455). The adjusted p-value was 0.421.

The total number of mutations in Hispanics appeared to be less, with a mean number of 1, compared to a mean number of 1.17 for non-Hispanics (relative risk 0.85 [95% CI=0.64-1.14; p=0.284]) by univariate analysis (Suppl Fig 2). When we performed multivariate analysis for age along with Hispanic status the relative risk for Hispanic was 0.90 (95% CI=0.66-1.23; p=0.496).

We also examined for associations between ethnicity and mutation class [cite Papaemmanuil NEJM]. The most common mutation classes were driver not class defining (24%), TP53/aneuploidy (22%), and mutated chromatin/splicing (21%) (Table 3). Favorable mutations NPM1/CEBPA/t(8;21)/inv(16) were observed in 18% of patients. There was a trend towards non-Hispanics being more likely to have a favorable mutation compared to Hispanics at 12.1% vs. 87.9%, respectively (p=0.084). Adverse risk fusions MLL/inv(3)/t(6;9) were seen in 4.4% of patients and, although rare, were more common in Hispanic patients compared to non-Hispanics (70% vs. 30%, p=0.004), and this remained significant after multiplicity adjustment (p=0.024).

For Review Only

Patient Outcomes

Full Cohort

With a median follow-up of 24.4 months (range, 0.2-147), 125 patients were still alive (55.6%): Thirty-five Hispanic patients (60.3%) and 90 non-Hispanic patients (53.9%). There was no difference in follow-up between the two groups (p=0.443). There was no difference in OS between Hispanics and non-Hispanics (p=0.128, Figure 1), although numerically Hispanics had improved OS at 12, 24, and 36 months, with the curves converging at 48 months. The 1-year OS for Hispanic patients was 85.6% and for non-Hispanic patients was 68.7% (p=0.589), and 3-year OS for Hispanic patients was 63.0% vs. 39.1% for non-Hispanic patients (p=0.735). The univariate HR for survival for non-Hispanics was 1.43 (95% CI=0.9-2.29; p=0.13). In multivariate analysis considering age, sex, ECOG, cytogenetic risk, and institution, the HR for survival for non-Hispanics was 1.15 (95% CI=0.69-1.90; p=0.594). Sixteen Hispanic patients (27.6%) underwent allogeneic hematopoietic stem cell translation (HSCT), compared to 36 non-Hispanic patients (21.7%, p=0.37).

De Novo Patients

In patients who were newly diagnosed (105 patients), OS was significantly better for Hispanic patients compared to non-Hispanics, at a median of 44.7 months vs. 14 months (log-rank test p-value=0.026; HR=0.46 [95% CI=0.23 to 0.92], p=0.0292, Figure 2), which is partially explained by Hispanics being younger at diagnosis than non-Hispanics (all patients, p=0.0013; newly diagnosed group, p=0.0529). In multivariate analysis accounting for age, sex, ECOG, and treating institution, this difference in survival persisted, but was no longer significant (HR=0.66 [95% CI=0.32 to 1.35], p=0.2573).

R/R Patients

Among the 101 patients with relapsed/refractory disease, there was no difference in OS by univariate or multivariate analysis (median 37.7 months for Hispanics, 30.9 months for non-Hispanics, HR=0.99 [95% CI=0.49 to 2.01], p=0.986 for UVA and HR=0.97 [95% CI=0.45 to 2.09], p=0.933 for MVA, Figure 2).

Patient Outcomes by Molecular Subgroups

We also examined OS by the most common class-defining molecular subgroups: Driver not class-defining, Mutated chromatin/splicing, NPM1/CEBPA/t(8;21)/inv(16), and TP53/aneuploidy.[6] Patients with TP53/aneuploidy had inferior OS compared to the other 3 mutation sub-categories (p=0.183) (Figure 3). There was no difference in survival in Hispanic vs. non-Hispanic patients with TP53/aneuploidy, Mutated chromatin splicing, or NPM1/CEBPA/t(8;21)/inv(16) (p=0.943) (Figure 4). However, Hispanic patients with Driver not class-defining mutations had improved OS at 100% vs. 62.9% at 1 year

For Review Only

(p<.0001) and 83.1% vs. 34.3% at 3 years (p=0.072) (Figure 4). In the Driver not class-defining group, there were 14 total patients with IDH mutations, 12 non-Hispanic and 2 Hispanic (p=0.008). The 2 IDH mutations occurring in Hispanic patients were both IDH2 R140. When we examined splicing factor mutations as a group (SF3B1, SRSF2, U2AF1, and ZRSR2), while the frequency was not different by ethnicity, OS was significantly better in non-Hispanic patients (p=0.010) (Suppl Fig 3). The frequency of RAS pathway mutations between ethnicity groups was also not different, although OS favored non-Hispanic patients (p=0.057) (Suppl Fig 4).

Discussion

From a healthcare perspective, racial and ethnic minorities in the United States broadly encompass Hispanics, African Americans or Blacks, American Indians and Alaska Natives, Asians, Native Hawaiians and Pacific Islanders according to the National Institute on Minority Health and Health Disparities (NIMHD)[7]. The Hispanic population collectively constitutes the largest minority group in the United States and currently represent approximately 18% of the US population[3,4]. This group includes people of Mexican, South/Central American, Cuban, Puerto Rican, or other Spanish-speaking cultures with half of the current Hispanic population residing in 3 states: Florida, Texas and California[8,9]. There are data to suggest that while the incidence of AML, barring APL, is lower in Hispanics compared to non-Hispanics, they are often diagnosed at a younger age and may have a poorer age adjusted overall survival[5,10-13]. The genetic and socioeconomic differences accounting for the possible discrepancies in prognosis and survival are not well accounted for at this time.

To better answer these questions, we examined NGS data on 225 AML patients and found that certain gene mutations clustered by ethnicity. IDH1 mutations were significantly more common in non-Hispanic patients, while WT1 mutations, which both effect TET protein activity and DNA methylation, were more common in Hispanic patients. There was no association between TET2 or IDH2 mutation frequency, or any other single gene mutation, and ethnicity. We also examined mutation risk groups and found that Hispanic patients were more likely to have an adverse risk AML fusion [11q23, inv(3), t(6;9)], and non-Hispanic patients were more likely to have a favorable risk fusion/mutation [t(8;21), inv(16), NPM1, CEBPA]. The strongest association was between Hispanic ethnicity and adverse risk AML fusions, which remained statistically significant after multiplicity adjustment (adjusted p-value=0.024). FLT3-ITD and TKD mutations were underrepresented in this cohort for unclear reasons. In non-Hispanics this may have been due to older age at presentation. In Floridian Hispanics FLT3 mutations were relatively uncommon (ITD in 7.6% and TKD in 1.7%).

We acknowledge that our study included all newly presenting AML patients, and that 19 (8.4%) of these patients were in complete remission after initially receiving treatment at

For Review Only

outside centers. These patients were included in analysis of mutations since pre-leukemic mutations often persist in remission. However, driver mutations may have been missed in these patients, and decreased the rate of detection of NPM1 and FLT3 mutations, for example.

Interestingly, Hispanic patients had improved OS compared to non-Hispanics, although the curves converged over time at around 4 years (p=0.128). When we examined only newly diagnosed patients, Hispanic patients had a significant and lasting improvement in OS (p=0.026). These observations are despite Hispanic patients appearing to be enriched for prognostically adverse cytogenetic abnormalities, particularly high-risk fusions such as MLL-r. When we performed multivariate analysis accounting for age, sex, ECOG, cytogenetic risk, and treating institution, this difference in OS was no longer observed, likely due to Hispanics being younger at time of AML diagnosis than non-Hispanics. Whether this age difference at AML diagnosis is due to the demographics of Florida versus differences in risk factors or disease biology remains to be discovered. Hispanic patients were also more likely to have de novo rather than secondary AML. At the minimum, and contrary to prior reports, our study suggests that Hispanic patients have *equivalent* age-adjusted OS compared to non-Hispanics, particularly if they are treated at an academic medical center. This may be due to the equalizing of socioeconomic disparities that can contribute to more advanced presentation, inadequate follow-up, and lack of availability of allogeneic stem cell transplantation. There was no difference in the rate of allogeneic transplantation in different ethnic groups on our study.

We sought to further assess survival differences in Hispanics and non-Hispanics with AML by examining distinct, class-defining molecular subgroups. In the entire cohort, patients with TP53/aneuploidy, regardless of ethnicity, had the poorest outcomes, although there was a tail on the curve with 10-15% long-term survival. Interestingly, Hispanic patients in the Driver not-class defining category had improved OS relative to non-Hispanics. One possible explanation for this difference is that non-Hispanic patients were enriched for mutations involved in cellular metabolism, suggesting that the heterogeneous Driver not-class defining category could be sub-divided by the presence or absence of mutations in IDH1/2. Hispanic patients did not have inferior OS in any molecular subgroup. However, when we looked at specific mutation classes, Hispanic patients with RAS pathway or RNA splicing factor mutations had inferior OS compared to non-Hispanics (p=0.057 and p=0.010, respectively).

This work sought to identify biologic discrepancies between Hispanic and non-Hispanic patients diagnosed with AML, which is not well characterized. Data from a Mexican AML population suggested, CEBPA, RUNX1 GATA2, TET2, AML1-ETO, U2AF1, ASXL1 and KIT are enriched in that population, whereas FLT3-ITD, DNMT3A, NPM1 and IDH2 were under-represented[14]. Our data of Floridian patients collected at 2 large academic centers showed 2 stand out differences on NGS; IDH1 mutations were underrepresented and

For Review Only

WT1 mutations were enriched in Hispanic patients compared to non-Hispanics, with a possibly lower overall mutational burden in Hispanic AML patients (possibly explained by younger age at presentation). Looking at mutation groups, there was a trend towards more adverse risk fusions and fewer favorable risk mutations in Floridian Hispanics; however, the rate of secondary AML was higher in non-Hispanics (which may be associated with age at presentation). In terms of the impact of specific mutation groups on outcomes, Hispanics patients without a Class defining driver mutation had improved OS compared to non-Hispanics, while those with a RAS pathway or splicing factor mutation had inferior OS.

In this study, after adjustment for age and other confounders, there was no significant difference in OS between Hispanic and non-Hispanic patients. That is to say, *when receiving equivalent care at an academic cancer center, overall survival may be comparable between Hispanic and Non-Hispanic patients, despite any molecular differences associated with ethnicity.*

There are some limitations to this study. Sample size in this study is limited and only includes those who geographically, and financially, could seek care at an academic center. This may in turn inherently exclude those with the worst SES and everything that comes along with it such as environmental exposures and other health stressors. Prior work as shown that regional location can play a role in AML outcomes in the Hispanic population[15]. Factors such as poverty, income and education status have also been linked to inferior survival, which may explain the inferior risk reported in SEER Medicare analysis[5,12,16-18]. Our data may suggest that, while there are some genetic differences, 'modifiable' factors such as socioeconomic inequity, healthcare access, environmental factors, etc are the primary drivers for the discrepancies in outcome reported in the past, and correction of these factors could improve AML outcomes in Hispanic patients. Secondly, the Hispanic diaspora is diverse, and includes people of Mexican, South/Central American, Cuban, Puerto Rican, or other Spanish-speaking cultures. Ethnicity on this study was self-reported, so we do not know the exact descent which is quite vast for Hispanics and may drive genetic differences. While Florida is one of 3 states where half the Hispanic population currently resides (the others being California and Texas), it likely over-represents certain subsets of the Hispanic population, such as Cuban or Puerto Rican Americans who are genetically disparate from other under-represented groups, such as those of Mexican origin[10,11]. Strengthening these data to further characterize the relationship between ethnicity and AML genotype and outcomes will require a multi-institutional effort moving forward.

1. Pollyea, D; Kohrt, HE; Yang, J; Chang, E; Gomez, S; Clarke, C. Actue leukemia in adult Hispanic Americans: differences in incidence rates by nativity. *Cancer Prevention &Current REsearch* 2014, 1 (1): 14-19.

2. Douer, D; Presont-Martin, S; Chang, E; Nichols, P; Watkins, K; Levine A. High frequency of acute promyelocytic leukemia among Latinos with acute myeloid luekemia. *Blood* **1996,** 1; 87 (1): 308-13.

3. Shoag, J; Barredo, J; Lossos, I; Pinheiro, P. Acute lymphoblastic leukemia mortality in Hispanic Americans. *Leukemia Lymphoma* **2020,** 61 (11): 2674-2681.

4. Swords, R.; Sznol, J.; Elias, R.; Watts, J.; Zelent, A.; Martin, E.; Vargas, F.; Bethel-Ellison, S.; Kobetz, E., Acute leukemia in adult Hispanic Americans: a large-population study. *Blood Cancer Journal* **2016,** *6* (10), e484-e484.

5. Darbinyan, K.; Shastri, A.; Budhathoki, A.; Helbig, D.; Snyder, R.; Pradhan, K.; Saleh-Esa, J.; Kornblum, N. S.; Binder, A. F.; Goel, S.; Janakiram, M.; Derman, O.; Gritsman, K.; Steidl, U.; Braunschweig, I.; Verma, A.; Mantzaris, I., Hispanic ethnicity is associated with younger age at presentation but worse survival in acute myeloid leukemia. *Blood advances* **2017,** *1* (24), 2120-2123.

6. Papaemmanuil E, Gerstung M, Bullinger L, Gaidzik VI, Paschka P, Roberts ND, Potter NE, Heuser M, Thol F, Bolli N, Gundem G, Van Loo P, Martincorena I, Ganly P, Mudie L, McLaren S, O'Meara S, Raine K, Jones DR, Teague JW, Butler AP, Greaves MF, Ganser A, Döhner K, Schlenk RF, Döhner H, Campbell PJ. Genomic Classification and Prognosis in Acute Myeloid Leukemia. N Engl J Med. 2016 Jun 9;374(23):2209-2221. doi: 10.1056/NEJMoa1516192.

7. Alvidrez, J.; Castille, D.; Laude-Sharp, M.; Rosario, A.; Tabor, D., The National Institute on Minority Health and Health Disparities Research Framework. *Am J Public Health* **2019,** *109* (S1), S16-S20.

8. Prevention, C. f. D. C. a., CDC Fast Facts: Health of Hispanic or Latino Population. **2021**.

9. Elizabeth Arias, P. D., Betzaida Tejada-Vera, M.S., Farida Ahmad, M.P.H., Kenneth D. Kochanek, M.A., Provisional Life Expectancy Estimates for 2020 from U.S. Department of Health and Human Services , Centers for Disease Control and Prevention, National Center for Health Statistics , National Vital Statistics System. *CDC: Vital Statistics Rapid Release* **2021,** *Report No. 015*.

10. Humes, K. R.; Jones, N. A.; Ramirez, R. R. *Overview of race and Hispanic origin: 2010*; 2010 Census Briefs, C2010BR-02; U.S. Department of Commerce, Economics and Statistics Administration, U.S. Census Bureau: Washington, DC, 2011.

11. Society, A. C., Cancer Facts and Figures: Hispanics/ Latinos 2018-2020. *American Cancer Society Surveillance Research* **2020**.

12. Kirtane, K.; Lee, S. J., Racial and ethnic disparities in hematologic malignancies. *Blood* **2017,** *130* (15), 1699-1705.

For Review Only

13. Patel, M. I.; Ma, Y.; Mitchell, B. S.; Rhoads, K. F., Understanding disparities in leukemia: a national study. *Cancer Causes & Control* **2012,** *23* (11), 1831-1837.
14. Gomez-Arteaga, A.; Mencia-Trinchant, N.; Tovar, A. M.; Carrillo, I. O.; Laguna, A. G.; Fuller, E. R.; Penafiel, C. R.; Guzman, M. L.; Hassane, D. C., Abstract C061: Molecular landscape of acute myeloid leukemia in Mexico. *Cancer Epidemiology, Biomarkers & Prevention* **2020,** *29* (6_Supplement_1), C061-C061.
15. Bencomo-Alvarez, A. E.; Gonzalez, M. A.; Rubio, A. J.; Olivas, I. M.; Lara, J. J.; Padilla, O.; Orazi, A.; Corral, J.; Philipovskiy, A.; Gaur, S.; Mulla, Z. D.; Dwivedi, A. K.; Eiring, A. M., Ethnic and border differences on blood cancer presentation and outcomes: A Texas population-based study. *Cancer* **2021,** *127* (7), 1068-1079.
16. Hong, S.; Brazauskas, R.; Hebert, K. M.; Ganguly, S.; Abdel-Azim, H.; Diaz, M. A.; Beattie, S.; Ciurea, S. O.; Szwajcer, D.; Badawy, S. M.; Gratwohl, A. A.; LeMaistre, C.; Aljurf, M. D. S. M.; Olsson, R. F.; Bhatt, N. S.; Farhadfar, N.; Yared, J. A.; Yoshimi, A.; Seo, S.; Gergis, U.; Beitinjaneh, A. M.; Sharma, A.; Lazarus, H.; Law, J.; Ulrickson, M.; Hashem, H.; Schoemans, H.; Cerny, J.; Rizzieri, D.; Savani, B. N.; Kamble, R. T.; Shaw, B. E.; Khera, N.; Wood, W. A.; Hashmi, S.; Hahn, T.; Lee, S. J.; Rizzo, J. D.; Majhail, N. S.; Saber, W., Community health status and outcomes after allogeneic hematopoietic cell transplantation in the United States. *Cancer* **2021,** *127* (4), 609-618.
17. Østgård, L. S. G.; Nørgaard, M.; Medeiros, B. C.; Friis, L. S.; Schoellkopf, C.; Severinsen, M. T.; Marcher, C. W.; Nørgaard, J. M., Effects of Education and Income on Treatment and Outcome in Patients With Acute Myeloid Leukemia in a Tax-Supported Health Care System: A National Population-Based Cohort Study. *Journal of Clinical Oncology* **2017,** *35* (32), 3678-3687.
18. Abraham, I. E.; Rauscher, G. H.; Patel, A. A.; Pearse, W. B.; Rajakumar, P.; Burkart, M.; Aleem, A.; Dave, A.; Bharadwaj, S.; Paydary, K.; Acevedo-Mendez, M.; Goparaju, K.; Gomez, R.; Carlson, K.; Tsai, S. B.; Quigley, J. G.; Galvin, J. P.; Zia, M.; Larson, M. L.; Berg, S.; Stock, W.; Altman, J. K.; Khan, I., Structural racism is a mediator of disparities in acute myeloid leukemia outcomes. *Blood* **2022,** *139* (14), 2212-2226.

For Review Only

Tables and Figures

Table 1. Patient demographics and disease characteristics.

Variable		*All*		*Hispanic*		*Non-Hispanic*		
		N	*%*	*N*	*%*	*N*	*%*	*p-value*
All		225	100.0	58	100.0	167	100.0	
Institution								0.020
	Moffitt	83	36.9	14	24.1	69	41.3	
	UM	142	63.1	44	75.9	98	58.7	
Gender								0.190
	Female	96	42.7	29	50.0	67	40.1	
	Male	129	57.3	29	50.0	100	59.9	
Race								<.0001
	White	195	86.7	48	82.8	147	88.0	
	Black	18	8.0	1	1.7	17	10.2	
	Other	12	5.3	9	15.5	3	1.8	
Median age (range)		65 (19-91)		60 (21-85)		67 (19-91)		0.001
Age at diagnosis								0.019
	<=60yos	88	39.1	31	53.4	57	34.1	
	61-70yos	67	29.8	16	27.6	51	30.5	
	>70yos	70	31.1	11	19.0	59	35.3	
ECOG								0.930
	Missing	8	3.6	6	10.3	2	1.2	
	ECOG:0	99	44.0	24	41.4	75	44.9	
	ECOG:1-3	118	52.4	28	48.3	90	53.9	
Subtype								0.003
	2 more	4	1.8	2	3.4	2	1.2	
	APL	1	0.4	1	1.7	.	.	
	Driver not class-defining	54	24.0	16	27.6	38	22.8	
	IDH2 R172	1	0.4	1	1.7	.	.	
	MLL/inv(3)/t(6;9)	10	4.4	7	12.1	3	1.8	
	Mutated chromatin/splicing	48	21.3	9	15.5	39	23.4	
	N/A	7	3.1	.	.	7	4.2	
	NPM1/CEBPA/T(8;21)/inv(16)	33	14.7	4	6.9	29	17.4	

Variable	All		Hispanic		Non-Hispanic		
	N	%	N	%	N	%	p-value
No driver	18	8.0	4	6.9	14	8.4	
TP53/aneuploidy	49	21.8	14	24.1	35	21.0	
Disease status							0.171
CR	19	8.4	8	13.8	11	6.6	
Newly Diagnosed	105	46.7	28	48.3	77	46.1	
Relapsed/Refractory	101	44.9	22	37.9	79	47.3	
Any mutations							0.554
No mutation	73	32.4	17	29.3	56	33.5	
One or more mutations	152	67.6	41	70.7	111	66.5	
Total number of mutations							
0	73	32.4	17	29.3	56	33.5	
1	85	37.8	28	48.3	57	34.1	
2	41	18.2	9	15.5	32	19.2	
3	19	8.4	4	6.9	15	9.0	
4	5	2.2	.	.	5	3.0	
5	2	0.9	.	.	2	1.2	
Overall survival							0.394
Alive	125	55.6	35	60.3	90	53.9	
Dead	100	44.4	23	39.7	77	46.1	

Table 2. Mutation frequency by ethnicity.

Mutation		All		Hispanic		Non-Hispanic		
		N	%	N	%	N	%	P-value*
All		225	100.0	58	100.0	167	100.0	
ASXL1								0.1434
	No	200	88.9	55	94.8	145	86.8	
	Yes	25	11.1	3	5.2	22	13.2	
CEBPA								1.00
	No	195	91.1	48	90.6	147	91.3	
	Yes	19	8.9	5	9.4	14	8.7	
DNMT3A								0.8456

Mutation		All		Hispanic		Non-Hispanic		
		N	%	N	%	N	%	P-value*
	No	184	81.8	47	81.0	137	82.0	
	Yes	41	18.2	11	19.0	30	18.0	
IDH1								0.0182
	No	204	90.7	57	98.3	147	88.0	
	Yes	21	9.3	1	1.7	20	12.0	
IDH2								0.6541
	No	195	86.7	49	84.5	146	87.4	
	Yes	30	13.3	9	15.5	21	12.6	
KIT								0.4279
	No	217	96.4	55	94.8	162	97.0	
	Yes	8	3.6	3	5.2	5	3.0	
NPM1								0.4547
	No	203	90.2	54	93.1	149	89.2	
	Yes	22	9.8	4	6.9	18	10.8	
PHF6								1.00
	No	207	92.0	54	93.1	153	91.6	
	Yes	18	8.0	4	6.9	14	8.4	
TET2								1.00
	No	185	82.2	48	82.8	137	82.0	
	Yes	40	17.8	10	17.2	30	18.0	
FLT3-TKD								0.6834
	No	217	96.4	57	98.3	160	95.8	
	Yes	8	3.6	1	1.7	7	4.2	
FLT3-ITD								1.00
	No	197	92.9	49	92.5	148	93.1	
	Yes	15	7.1	4	7.6	11	6.9	
MLL-PTD								0.3701
	No	205	96.7	50	94.3	155	97.5	
	Yes	7	3.3	3	5.7	4	2.5	

Mutaton		All N	All %	Hispanic N	Hispanic %	Non-Hispanic N	Non-Hispanic %	p-value
All		67	100.0	20	100.0	47	100.0	
BCOR								1.00
	No	61	91.0	18	90.0	43	91.5	
	Yes	6	9.0	2	10.0	4	8.5	
JAK2								0.6649
	No	60	89.6	19	95.0	41	87.2	
	Yes	7	10.4	1	5.0	6	12.8	
KRAS								0.0602
	No	61	91.0	16	80.0	45	95.7	
	Yes	6	9.0	4	20.0	2	4.3	
NF1								0.6604
	No	61	91.0	19	95.0	42	89.4	
	Yes	6	9.0	1	5.0	5	10.6	
NRAS								1.00
	No	60	89.6	18	90.0	42	89.4	
	Yes	7	10.4	2	10.0	5	10.6	
PDGFRB								0.6306
	No	62	92.5	18	90.0	44	93.6	
	Yes	5	7.5	2	10.0	3	6.4	
RUNX1								0.7597
	No	51	76.1	16	80.0	35	74.5	
	Yes	16	23.9	4	20.0	12	25.5	
SF3B1								1.00
	No	62	92.5	19	95.0	43	91.5	
	Yes	5	7.5	1	5.0	4	8.5	
SRSF2								0.7410
	No	55	82.1	16	80.0	39	83.0	
	Yes	12	17.9	4	20.0	8	17.0	
STAG2								0.1678
	No	61	91.0	20	100.0	41	87.2	
	Yes	6	9.0	*	*	6	12.8	

Mutaton		*All*		*Hispanic*		*Non-Hispanic*		
		N	*%*	*N*	*%*	*N*	*%*	*p-value*
TP53								0.6872
	No	59	88.1	17	85.0	42	89.4	
	Yes	8	11.9	3	15.0	5	10.6	
WT1								0.0455
	No	59	88.1	15	75.0	44	93.6	
	Yes	8	11.9	5	25.0	3	6.4	
KRAS, NRAS, and NF1								0.2756
	No	206	91.6	51	92.8	155	87.9	
	Yes	19	8.4	7	7.2	12	12.1	
SF3B1, SRSF2, U2AF1, or ZRSR2								1.00
	No	48	71.6	14	70.0	34	72.3	
	Yes	19	28.4	6	30.0	13	27.7	

*P-values were obtained from Chi-square test.

Table 3. AML molecular sub-categories by ethnicity.

Variable	*All*		*Hispanic*		*Non-Hispanic*		
	N	*%*	*N*	*%*	*N*	*%*	*p-value*
All	225	100.0	58	100.0	167	100.0	
Subtype							
2 more	4	1.8	2	3.4	2	1.2	-
APL	1	0.4	1	1.7	.	.	-
Driver not class-defining	54	24.0	16	27.6	38	22.8	0.573
IDH2 R172	1	0.4	1	1.7	.	.	-
MLL/inv(3)/t(6;9)	10	4.4	7	12.1	3	1.8	0.004
Mutated chromatin/splicing	48	21.3	9	15.5	39	23.4	0.285
N/A	7	3.1	.	.	7	4.2	-
NPM1/CEBPA/T(8;21)/inv(16)	33	14.7	4	6.9	29	17.4	0.084
No driver	18	8.0	4	6.9	14	8.4	0.937
TP53/aneuploidy	49	21.8	14	24.1	35	21.0	0.748

Figure 1. Overall survival by Ethnicity in all patients.

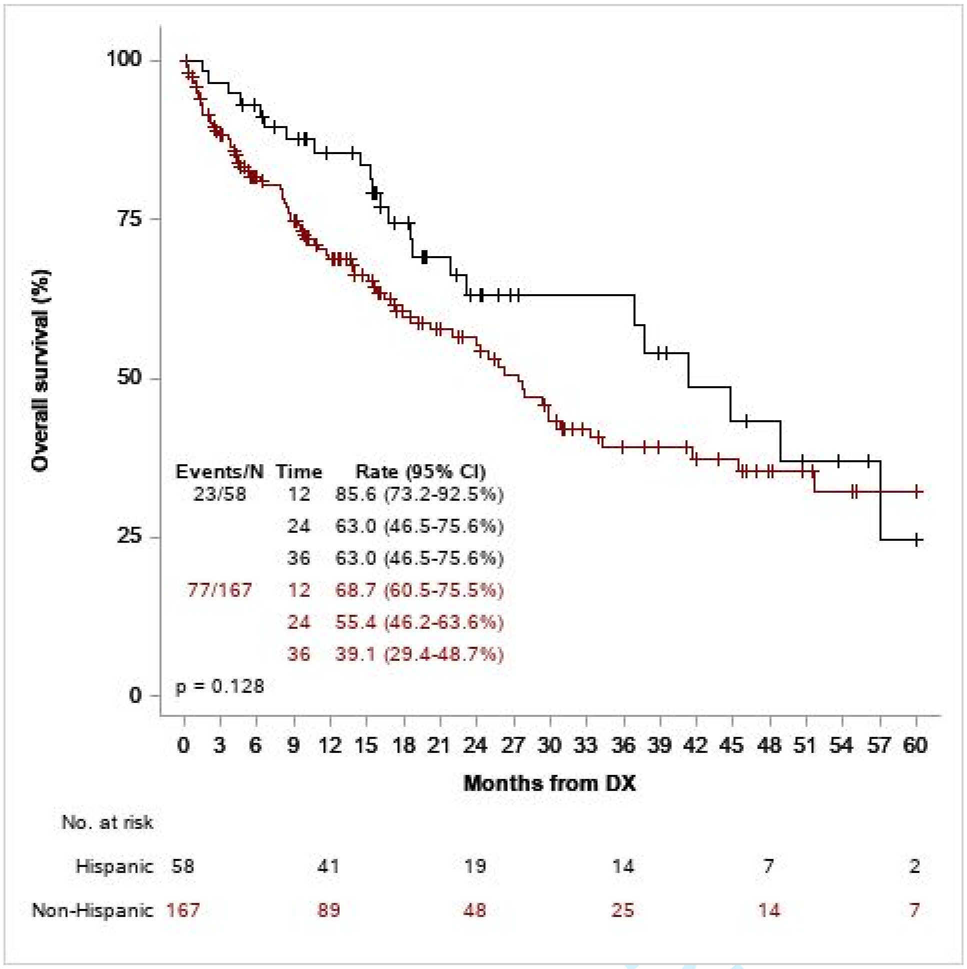

Figure 2. Overall survival in newly diagnosed patients and relapsed/refractory patients.

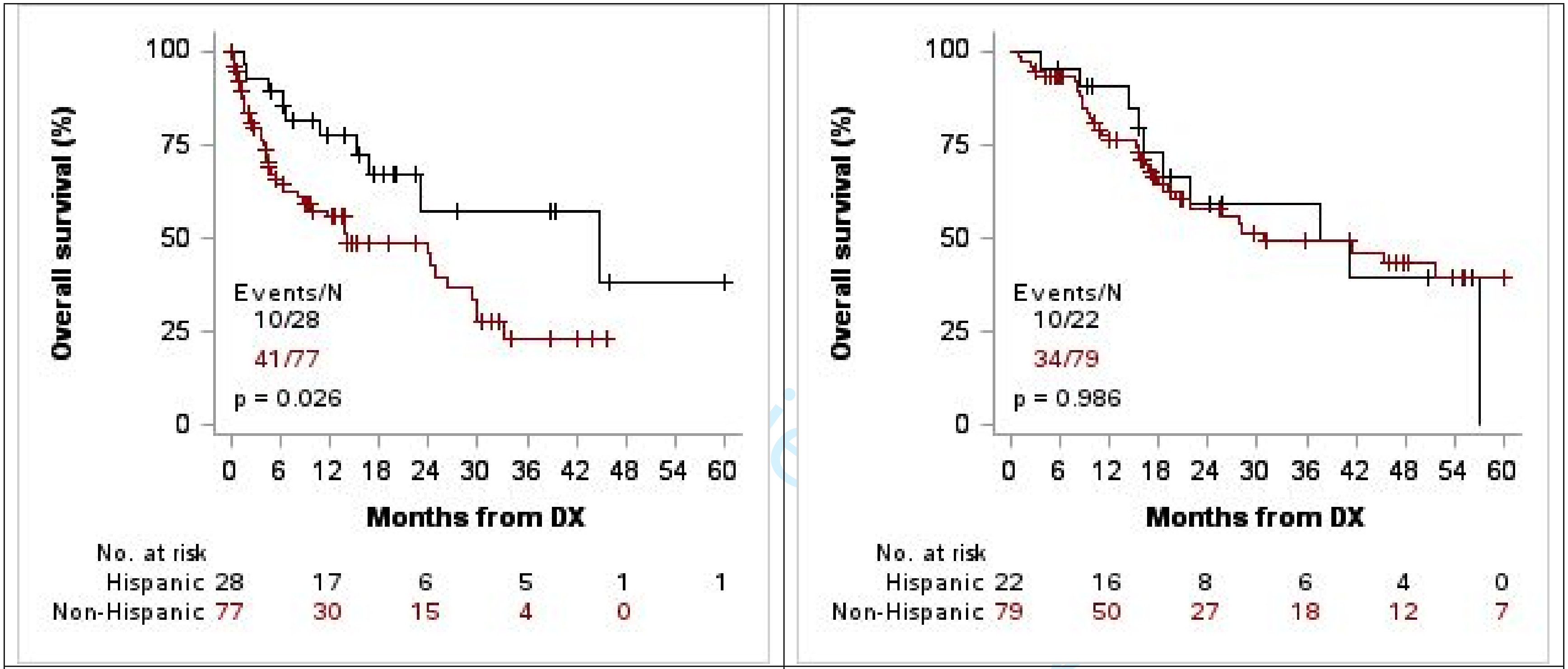

A. Newly diagnosed patients

B. Relapsed/Refractory patients

Figure 3. Overall survival by molecular subtype.

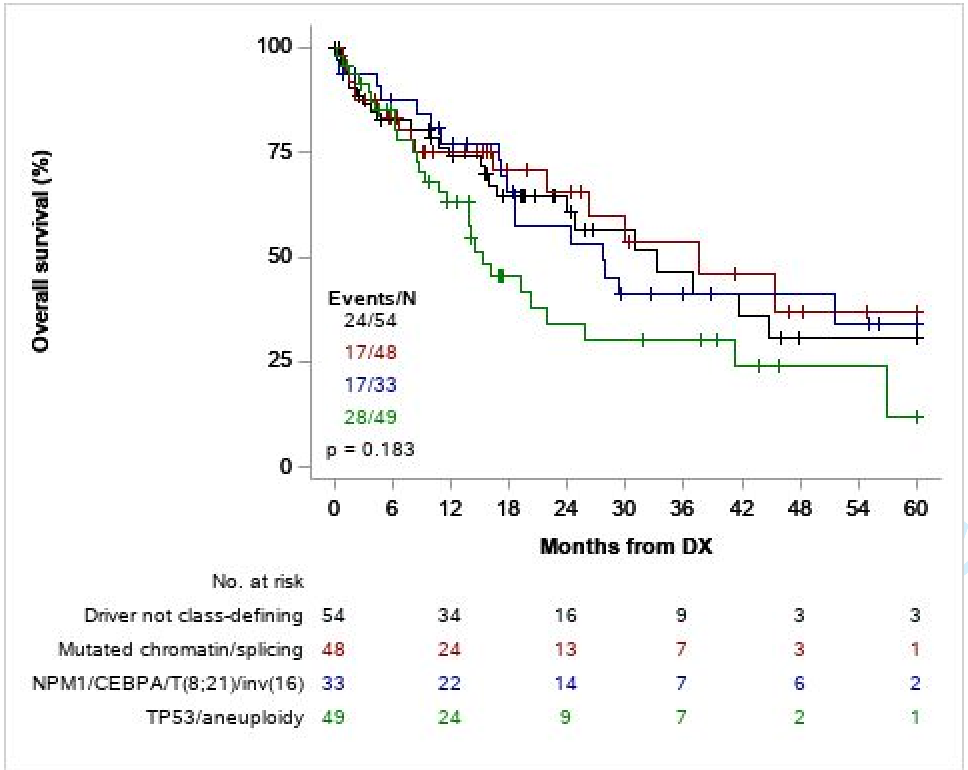

Figure 4. Overall survival for molecular subtypes by ethnicity.

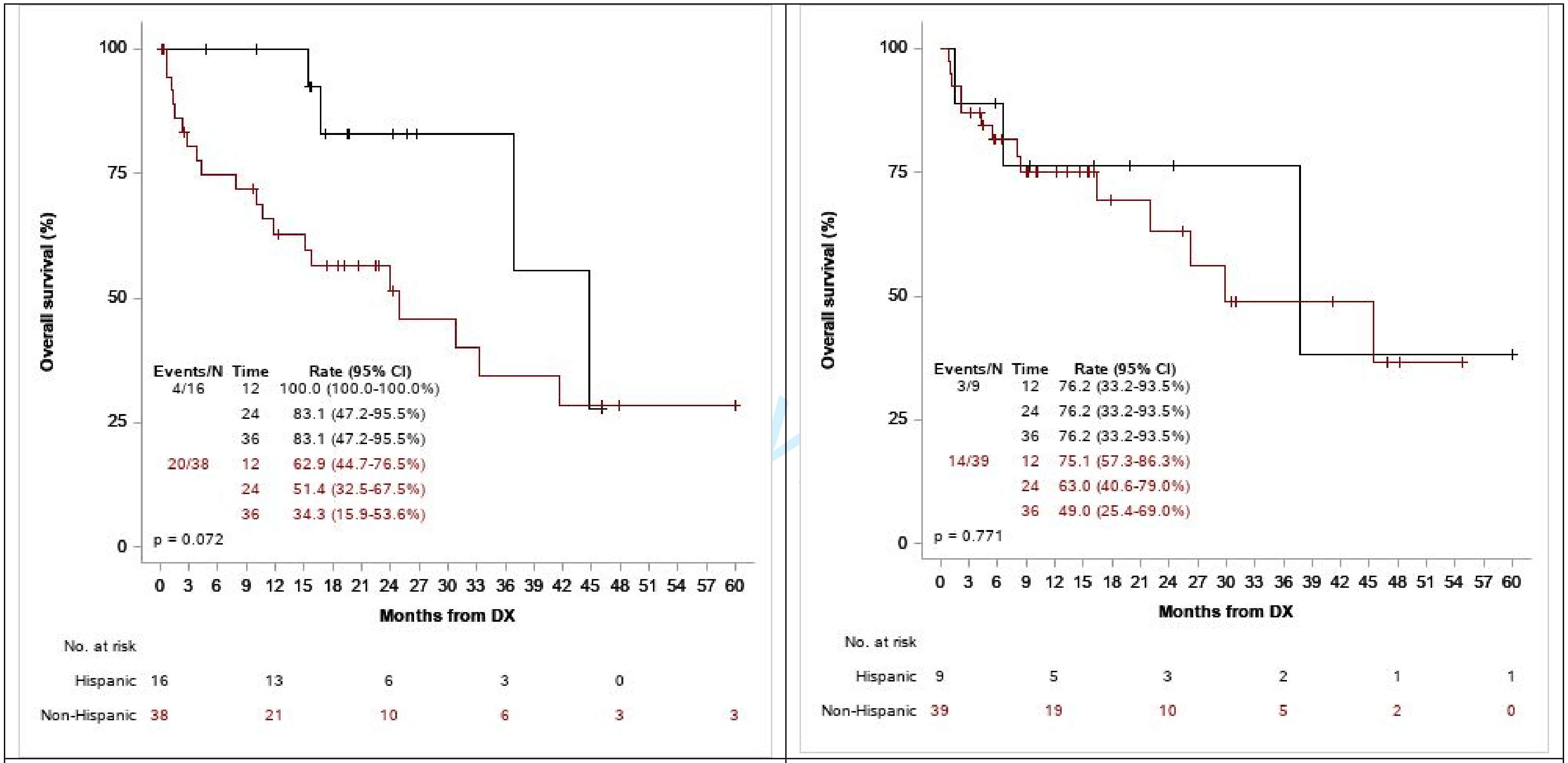

A. Driver not class-defining

B. Mutated chromatin/splicing

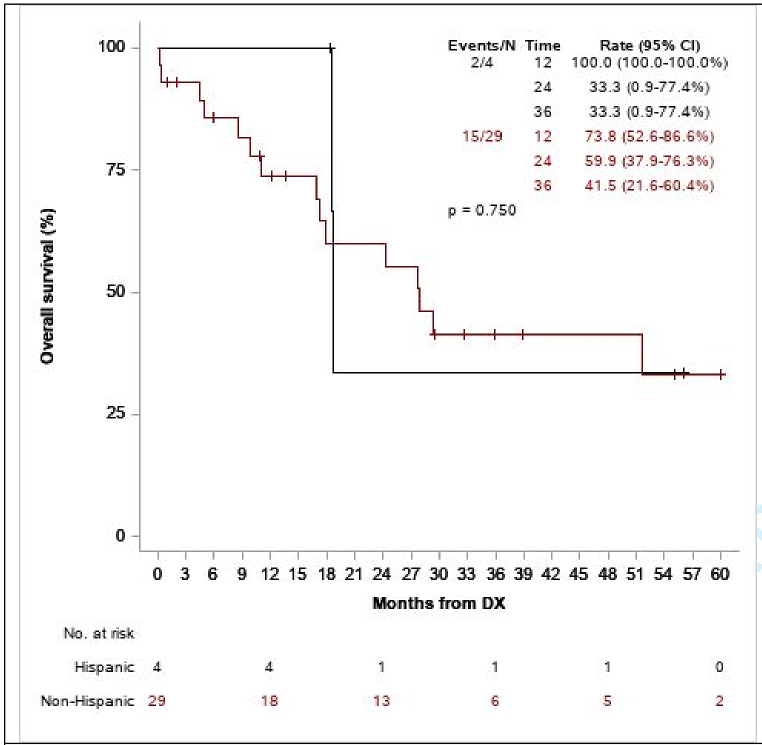

C. NPM1/CEBPA/T(8;21)/inv(16)

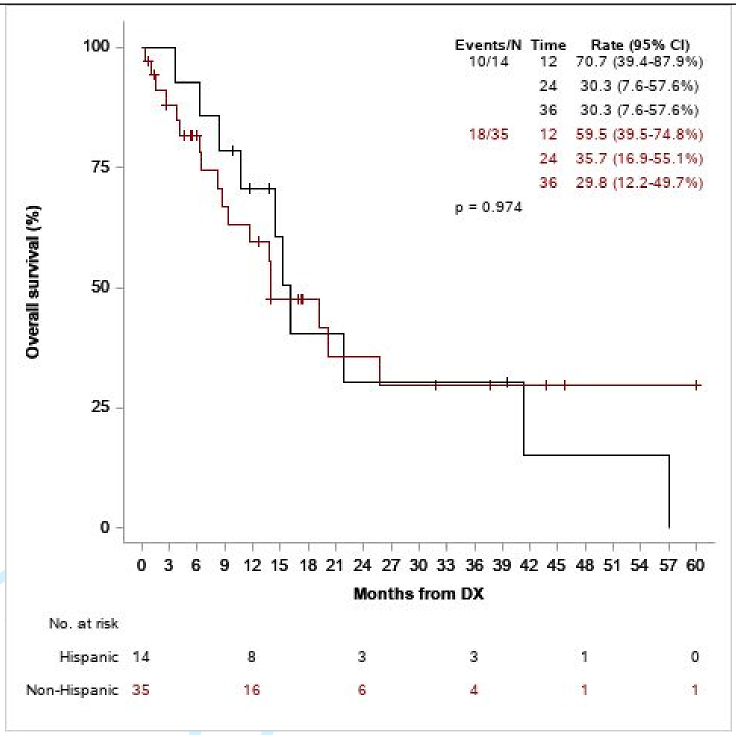

D. TP53/aneuploidy

Suppl Fig 1

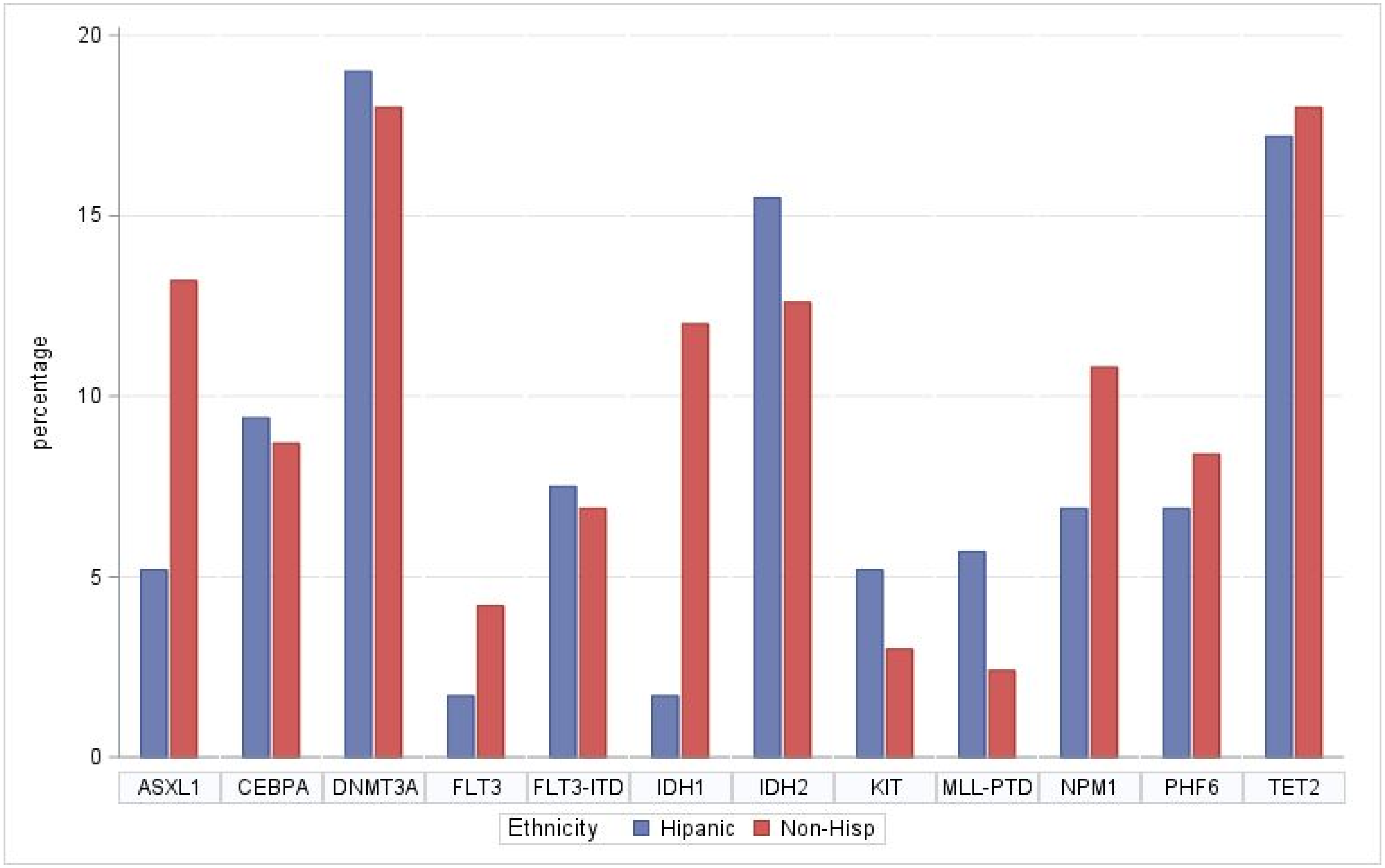
20
15
10
5
0
percentage
ASXL1
CEBPA
DNMT3A
FLT3
FLT3-ITD
IDH1
IDH2
KIT
MLL-PTD
NPM1
PHF6
TET2
Ethnicity
Hipanic
Non-Hisp

Suppl Fig 2

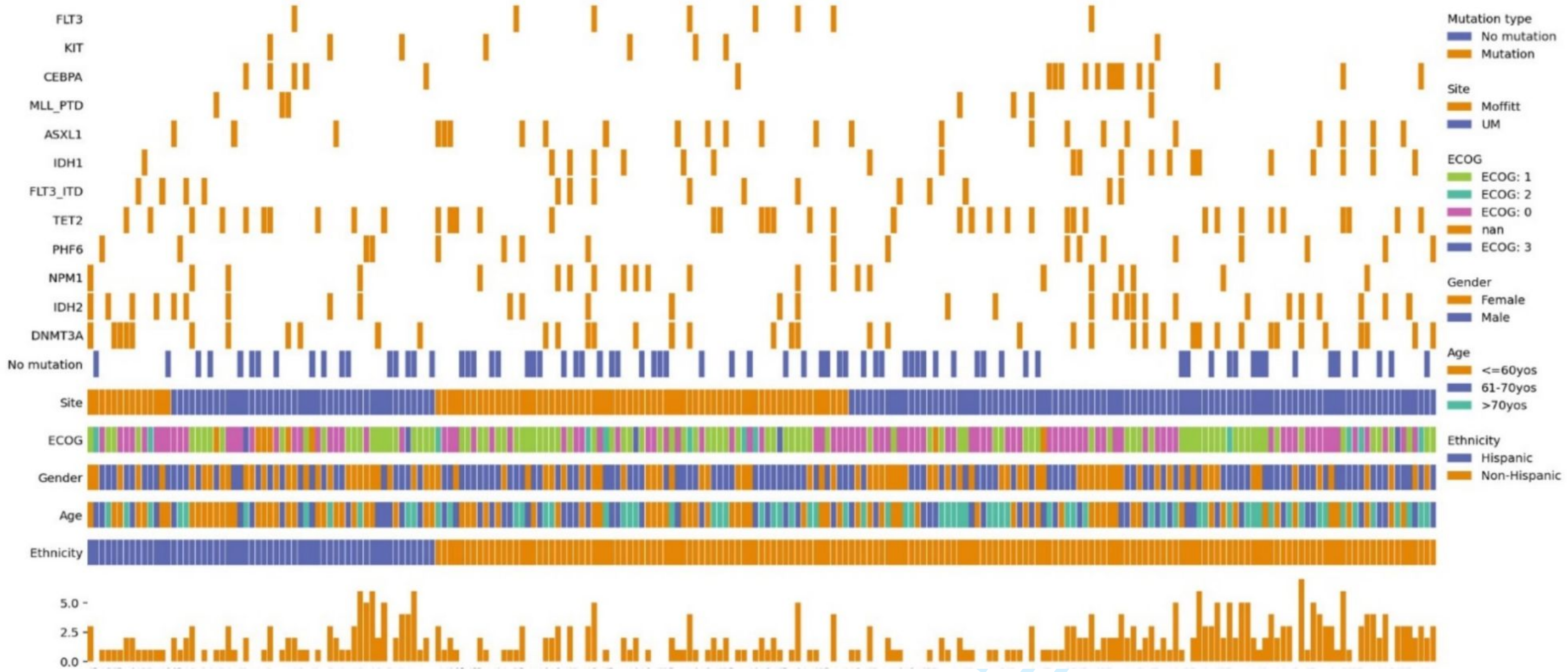
FLT3
KIT
CEBPA
MLL_PTD
ASXL1
IDH1
FLT3_ITD
TET2
PHF6
NPM1
IDH2
DNMT3A
No mutation
Site
ECOG
Gender
Age
Ethnicity
5.0
2.5
0.0
Mutation type
No mutation
Mutation
Site
Moffitt
UM
ECOG
ECOG: 1
ECOG: 2
ECOG: 0
nan
ECOG: 3
Gender
Female
Male
Age
<=60yos
61-70yos
>70yos
Ethnicity
Hispanic
Non-Hispanic

Suppl Fig 3

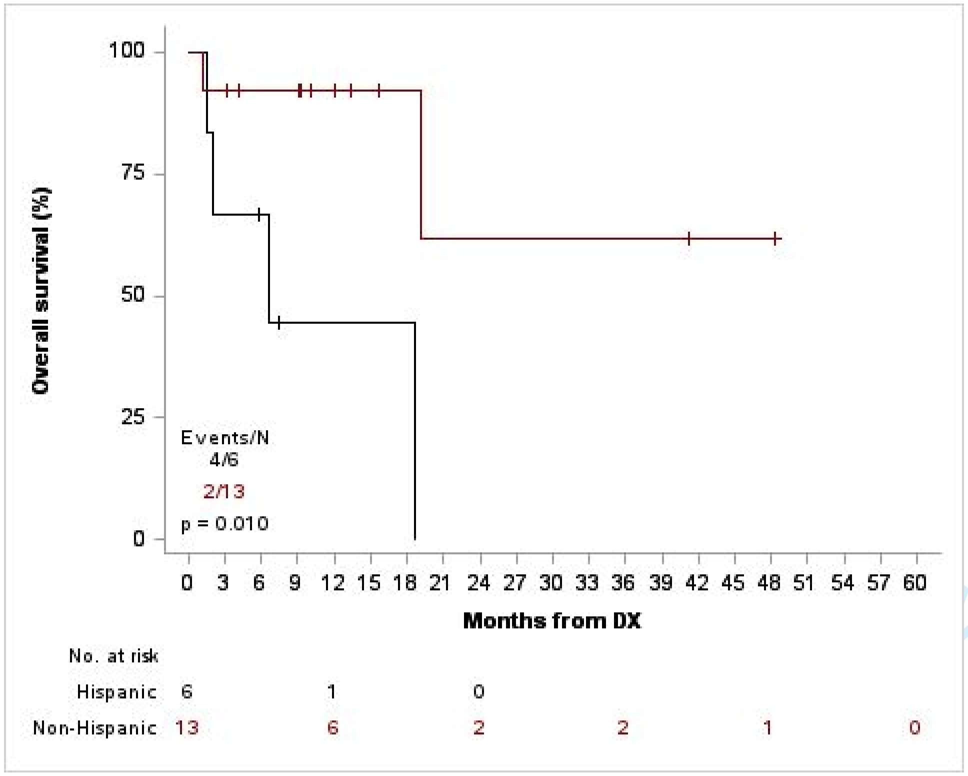

Mutation*:* SF3B1, SRSF2, U2AF1, or ZRSR2

Only

Suppl Fig 4

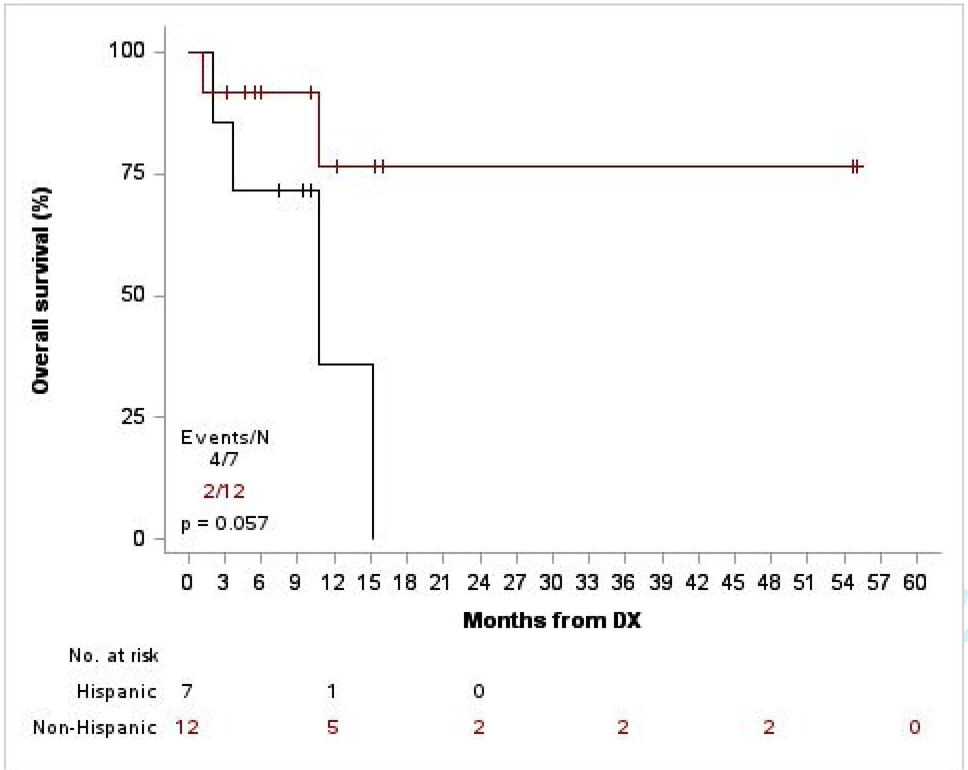

Mutation: KRAS, NRAS, and NF1

Only
